# Oocyte gene mutations increase rates of total fertilization failure: a systematic review and meta-analysis

**DOI:** 10.1186/s12958-026-01546-9

**Published:** 2026-04-18

**Authors:** Begüm Kepkep, Máté Szabolcs Botos, İpek Yazıcı, Lőrinc Frivaldszky, Anett Rancz, Péter Hegyi, Nándor Ács, Zsolt Melczer, Boglárka Szentes, Miklós Sipos

**Affiliations:** 1 Centre for Translational Medicine, Semmelweis University, Baross utca 22, Budapest, 1085 Hungary; 2 Department of Obstetrics and Gynecology, Semmelweis University, Budapest, Hungary; 3 Fejér County Szent György University Teaching Hospital, Székesfehérvár, Hungary; 4 MRE Bethesda Children’s Hospital, Budapest, Hungary; 5 Institute of Pancreatic Diseases, Semmelweis University, Budapest, Hungary; 6 Institute for Translational Medicine, Medical School, University of Pécs, Pécs, Hungary

**Keywords:** Total fertilization failure, Oocyte gene mutations, Assisted reproductive technology, Fertilization rate, Infertility genetics

## Abstract

**Introduction:**

Total fertilization failure (TFF) remains a critical barrier in assisted reproductive technology (ART), occurring in 5–10% of treatment cycles. While TFF can result from both male and female gamete factors, emerging evidence suggests that oocyte gene mutations may play a significant role. This study systematically reviews and meta-analyzes the influence of specific oocyte gene mutations (OGMs) on fertilization outcomes, focusing on their association with TFF and fertilization rates (FRs).

**Methods:**

This study has a preregistered protocol (CRD42024608566). A comprehensive search was conducted in PubMed, EMBASE, and Cochrane Library on December 10, 2024 and updated on February 5, 2026. Studies involving women undergoing ART with known oocyte gene mutations—including WEE2, PATL2, TUBB8, and others—were evaluated alongside studies of women without identified mutations. TFF and FR were the primary and secondary outcomes, respectively. Data synthesis included pooled proportions with confidence intervals (CIs) using random-effects models.

**Results:**

Of the 11,966 records identified, 105 studies were included. A total of 74 studies reported on women with 19 different OGMs, with 31 studies serving as indirect comparators. TFF rates were higher in women with specific mutations including *WEE2*,* PATL2*, and *TUBB8*, when analyzed alongside the indirect comparison patients; which were 66% (95% CI: 44–82%), 67% (95% CI: 50–81%) and 18% (95% CI: 8–38%) respectively. FRs were lower among carriers of these mutations: 3% (95% CI: 1–10%), 2% (95% CI: 0–10%) and 20% (95% CI: 10–38%) respectively. In contrast, the indirect comparator group had a low TFF rate, which was 9% (95% CI: 6%; 14%) and high FR, which was 67% (95% CI: 62%; 71%).

**Conclusion:**

Our findings suggest that specific OGMs may be associated with increased TFF rates and decreased FRs in ART. These results highlight the potential clinical value of genetic screening in women with unexplained infertility or repeated ART failures.

**Supplementary Information:**

The online version contains supplementary material available at 10.1186/s12958-026-01546-9.

## Key points

1. The presence of several oocyte gene mutations (OGMs) results in higher rates of total fertilization failure (TFF) and lower fertilization rates (FRs).

2. WEE2 and PATL2 mutations showed increased TFF rates up to 67% in assisted reproduction.

3. Physicians should consider genetic testing for OGM for women undergoing ART with a diagnosis of unexplained infertility.

**Supplementary Information:**

The online version contains supplementary material available at 10.1186/s12958-026-01546-9.

## Introduction

Infertility, defined as the inability to conceive after a year of unprotected sexual activity, affects couples worldwide at both physical and psychological levels [1]. Approximately 15% of couples worldwide experience infertility [2], highlighting the need for increased awareness and understanding within the medical and social spheres. There are several underlying causes, such as ovulatory dysfunction, male factor infertility, tubal diseases, and lastly, unexplained infertility [1].

The diagnosis of infertility can be a challenging and emotionally difficult experience for women to process [3]. In this context, assisted reproductive technology (ART) presents a viable option for those struggling with unexplained infertility, mild male factor infertility, and stage 1 and 2 endometriosis [4]. ART faces several complex challenges that need to be addressed. Treatment cycles are lengthy and expensive. The cost of ART depends on the protocol and medications used and varies worldwide, ranging from $2,109 to $18,592 [5].

Besides the psycho-social and economic burden, patients also face physical changes and adverse events during ART. Hyperstimulation, ovarian torsion, infection, and bleeding are possible adverse events of ART [6]. An unsuccessful attempt can result in considerable frustration and distress for both healthcare providers and women undergoing treatment [7].

Various factors can contribute to unsuccessful ART cycles, one of which is total fertilization failure (TFF) [8]. TFF is an unfortunate event observed in 5–10% of assisted reproduction cases [9]. It is defined as the failure of all oocytes to be fertilized in ART cycles [8]. In our study, we focus on the oocyte component of TFF.

Some mutations can disrupt normal maturation, leading to inadequate development or abnormalities that ultimately impede fertilization. Sang et al. (2021) summarize the effects of various oocyte mutations: PANX1 leads to oocyte death during the germinal vesicle (GV) stage, while PATL2 causes GV arrest. TUBB8 and TRIP13 result in metaphase I (M1) arrest, and WEE2, TLE6, and CDC20 contribute to fertilization failure by preventing the transition from metaphase II (M2) to the two pronuclear (PN) stages. Additionally, mutations such as PADI6, NLRP2, and NLRP5 can cause arrest in early embryonic development [10].

These mutations can be detected in various ways. One such method is DNA sequencing, which can be conducted using peripheral blood samples to identify mutations in oocytes [11]. This test is straightforward for both patients and physicians. However, the costs of whole-exome sequencing are prohibitively high, estimated at $555 to $5,169 per [9].

Considering the aforementioned points, we aimed to investigate differences in TFF rates and fertilization rates (FR) between women carrying specific oocyte gene mutations (OGMs) and non-genotyped indirect comparator patients.

## Methods

This work was conducted as part of the Systems Education Program at Semmelweis University [12].

We report our systematic review and meta-analysis in accordance with the Preferred Reporting Items for Systematic Reviews and Meta-Analyses (PRISMA) 2020 guidelines [13], and followed the Cochrane Handbook [14].

### Protocol deviation

The study protocol was registered with PROSPERO (registration number CRD42024608566). A minor deviation was made by including case reports and conference abstracts for the mutated cases, due to the limited number of studies with small sample sizes.

The inclusion of single-arm case reports and conference abstracts for the exposure group was necessitated by the current state of the literature on oocyte gene mutations. These mutations are infrequent (see the prevalence under eligibility criteria), recently characterized, and not routinely screened for in clinical ART populations, which has limited the feasibility of conducting extensive cohort studies or randomized controlled trials. As a result, the available evidence is confined mainly to descriptive case reports and preliminary conference communications.

In contrast, higher-level evidence, such as cohorts and randomized trials exists for the indirect comparator group, as fertilization outcomes in unselected ART populations have been extensively studied over several decades. The imbalance in evidence quality between groups, therefore, reflects differences in knowledge maturity and study feasibility rather than selective inclusion criteria. Excluding case reports and abstracts would have resulted in the omission of nearly all available data on mutation-positive patients, undermining the objective of characterizing the potential impact of these mutations on TFF and FR.

### Eligibility criteria

Our study indirectly compares TFFs and FRs in women undergoing assisted reproduction with OGM with those without such mutations. We used the PECO framework to design our research. The population (P) is women undergoing assisted reproduction; the exposure (E) is oocyte gene mutations, the (indirect) comparator (C) is non-genotyped oocyte gene mutations; and lastly, the primary outcome (O) is TFF and the secondary outcome is FR.

For the oocyte gene mutation arm, we included studies that assessed the mutations in TUBB8, PATL2, WEE2, NLRP2, NLRP5, BTG4, FBXO43, MEI1, MOS, OOEP, PADI6, PANX1, TLE6, ASTL, CDC20, CDC3, CDC23, APC13 and TRIP13. For the non-genotyped (indirect comparator) arm, we used articles that assessed the fertilization outcomes of different ARTs, such as in vitro fertilization (IVF) and intracytoplasmic sperm injection (ICSI). We were able to use articles that did not perform genetic testing for oocyte gene mutations, given the low prevalence of these mutations in the population. We determined the prevalence of various OGMs using allele frequencies sourced from genetic databases. Notable prevalence numbers include 0.002% for WEE2, 0.105% for PATL2, and 0.076% for TUBB8. The GnomAD genetic database [15] has been used to calculate of the allele frequencies of these OGMs globally. The lack of direct comparative studies in the literature regarding OGMs has led us to conduct this indirect comparison. However, we recognize that comparing mutation-positive cases with non-genotyped control groups presents a significant methodological limitation in our study.

### Information sources

**Fig. 1:**
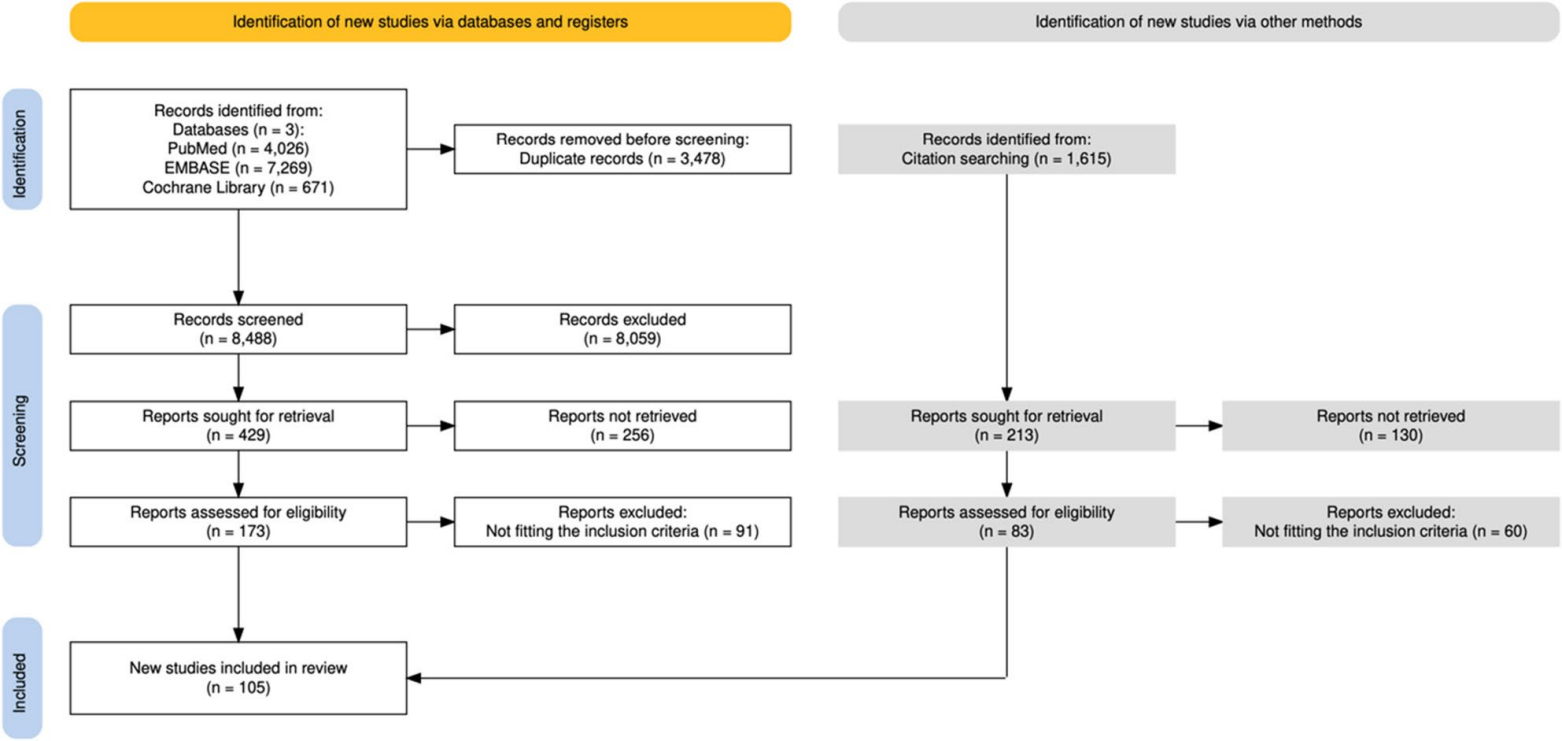
PRISMA flowchart presenting the article selection process

Our systematic search was conducted on December 10, 2024, in three databases: MEDLINE via PubMed, EMBASE, and Cochrane Library, and updated on February 5, 2026. Citation chasing was performed on February 20, 2025 to identify additional studies. The results of the search are shown in Fig. 1.

### Search strategy

The search key comprised two domains, one related to the population and the other to our outcome. The detailed search strategy is presented in the Supplementary Material, *Appendix S1*.

### Selection process

The article selection process was conducted by two independent review authors, BK and MSzB. Conflicts were resolved by IY as the third reviewer. Articles retrieved using the search key across three databases were downloaded into EndNote 21 [16], where we removed duplicate articles. The articles remaining after duplicate removal were uploaded to Rayyan [17] for title and abstract selection. After title and abstract selection, we moved on to full-text selection. Cohen’s kappa statistic was utilized to evaluate the level of agreement between raters throughout the selection and extraction processes.

### Data collection process

Data from the eligible articles were independently collected by two authors, BK and MSzB, and any discrepancies were resolved by IY.

### Data items

Data extraction elements differed between the exposure and control group articles. The following data were extracted from both the exposure and control groups: (1) first author, (2) the year of publication, (3) study population, (4) patient age and (5) study type. The following data were extracted specifically from the exposure group: (1) total number of patients with oocyte gene mutations, (2) total number of patients without oocyte gene mutations, (3) number of oocytes collected, and (4) number of fertilized oocytes. The following data were extracted specifically from the control group: (1) total number of patients, oocytes, or cycles, (2) number of TFF cases and (3) FR.

Some articles explicitly reported the number of TFF events, while others reported only the number of collected oocytes and fertilized oocytes. In these instances, we applied the same definition of TFF that we used for the articles in the exposure group.

### Study risk of bias assessment

The Joanna Briggs Institute (JBI) Checklists for case reports, case series, and prevalence studies were used to assess the risk of bias [18]. Two authors independently performed the risk of bias assessment. Any disagreements were resolved through discussion.

### Synthesis methods

The primary outcome, TFF calculation, was performed in cases where the articles did not report the number of TFFs. We defined a TFF event as a case where none of the collected oocytes were fertilized. We present one forest plot in which TFF occurrence was calculated based on cycles without oocyte fertilization; this is referred to as cycle-based (see Supplementary Material *Figure **S1*). In contrast, all other plots presented in the manuscript and the Supplementary Material relating to TFF were calculated based on patients without oocyte fertilization; these are referred to as patient-based. The articles in the indirect comparator group varied significantly in how they reported data. Since our study focused on TFF data from assisted reproduction cycles, we relied on various literature sources concerning ART. Some articles reported fertilization and TFF data by patient, while others reported them by cycle. Analyzing these different types of data reporting together would not be statistically appropriate. Therefore, we categorized them and analyzed each type using separate Forest plots.

Given considerable between-study heterogeneity in all cases, a random-effects model was used to pool effect sizes. Proportions were used as effect size measure with 95% confidence intervals (CI). To calculate study and pooled proportions, we extracted the number of patients and the number with the event of interest from each study.

A random-intercept logistic regression model was used to pool outcomes, as recommended by Schwarzer et al. [19] and Stijnen et al. [20]. The maximum likelihood method was used to estimate the heterogeneity variance measure (τ2).

In the forest plots, the Clopper-Pearson method was used to calculate the CI for proportions in individual studies. We summarized the findings of the meta-analysis presented in forest plots. Additionally, between-study heterogeneity was described by Higgins & Thompson’s I^2^ statistics [21, 22]. Small study publication bias was primarily assessed by visual inspection of Funnel-plots. Funnel plot asymmetry was tested by Peters’ test (modified Egger’s test) in case of outcomes including at least 10 studies, as recommended by Peters et al. [23] Potential outlier publications were explored using various influence measures and plots, following the recommendations of Harrer et al. [24]. All statistical analyses were conducted with R [25] using the meta package [26] for basic meta-analysis calculations and plots, and the dmetar package [27] for additional influential analysis calculations and plots.

## Results

### Search and selection

Our PRISMA flowchart is presented in Fig. 1 [28].

Altogether 11,966 articles were identified. We included 105 articles for data extraction. Cohen’s kappa values for title-abstract selection and full text selection were 0.91 and 0.86, respectively.

### Basic characteristics of included studies

The articles and data reporting for the indirect comparator group showed greater heterogeneity due to the unselected ART populations being used in the studies. Baseline characteristics of the included analyses are detailed in Supplementary Material *Table S1.*

### Primary outcome: TFF

We included 74 articles in the oocyte gene mutation group and 31 articles in the control indirect comparator group.

**Fig. 2:**
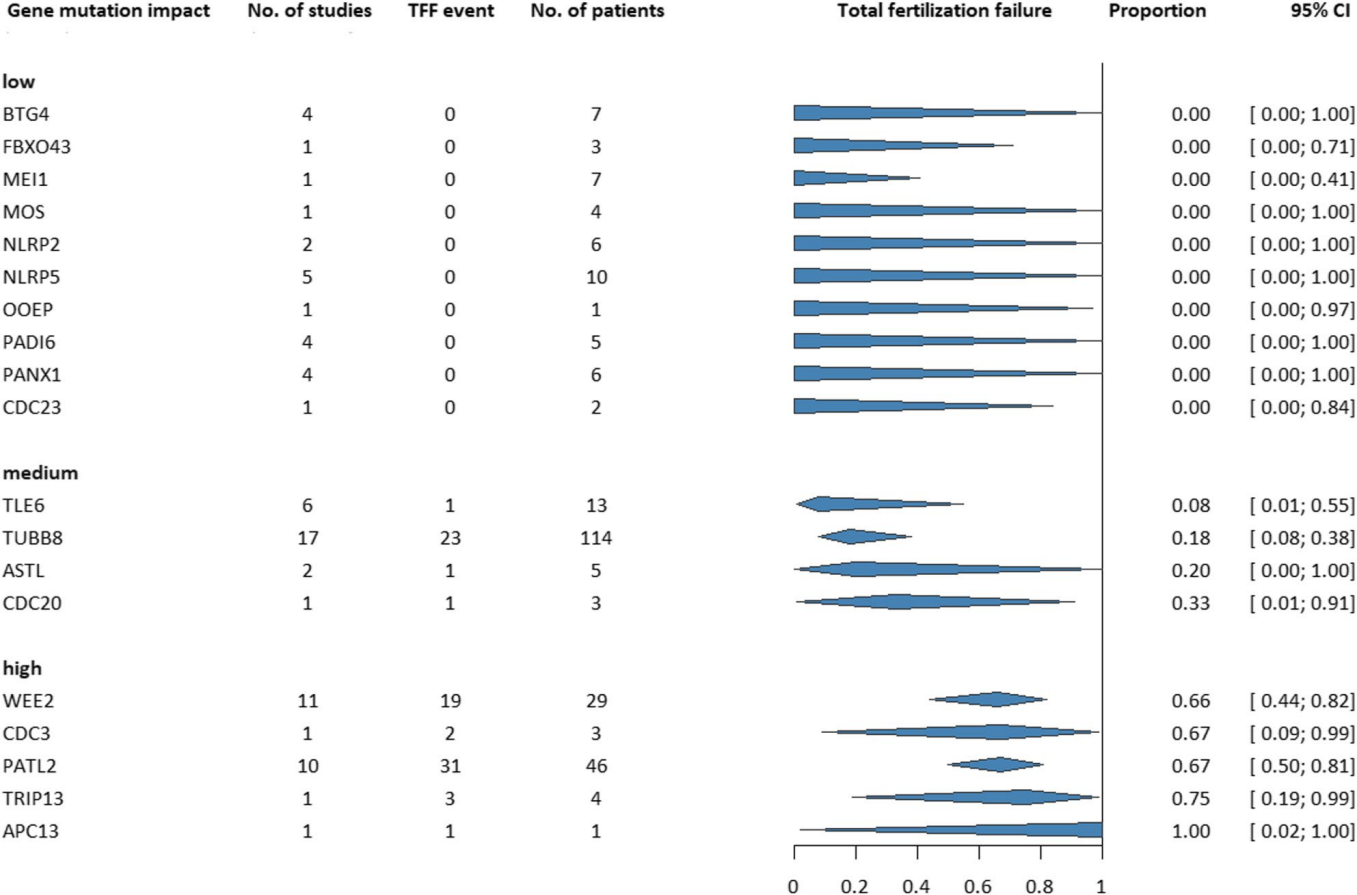
Summary plot of pooled proportion of TFF in patient-based studies for different types of OGMs grouped by magnitude of impact

TFF rates were increased due to several oocyte gene mutations, while some mutations did not affect the rates. Figure 2 shows the summary forest plot of TFF rates for 19 different OGMs, based on data of 74 studies involving 269 patients. Different types of mutations have different effects on the TFF rates, the magnitude of impact is grouped as low (0%), medium (between 0% and 50%) and high (50% and 100%). Among these, the highest TFF rates were observed in CDC3, PATL2, WEE2, APC13 and TRIP13 mutations. We investigated these OGMs in greater detail, however, only WEE2 and PATL2 mutations were eligible for separate investigation in a meta-analysis.

**Fig. 3:**
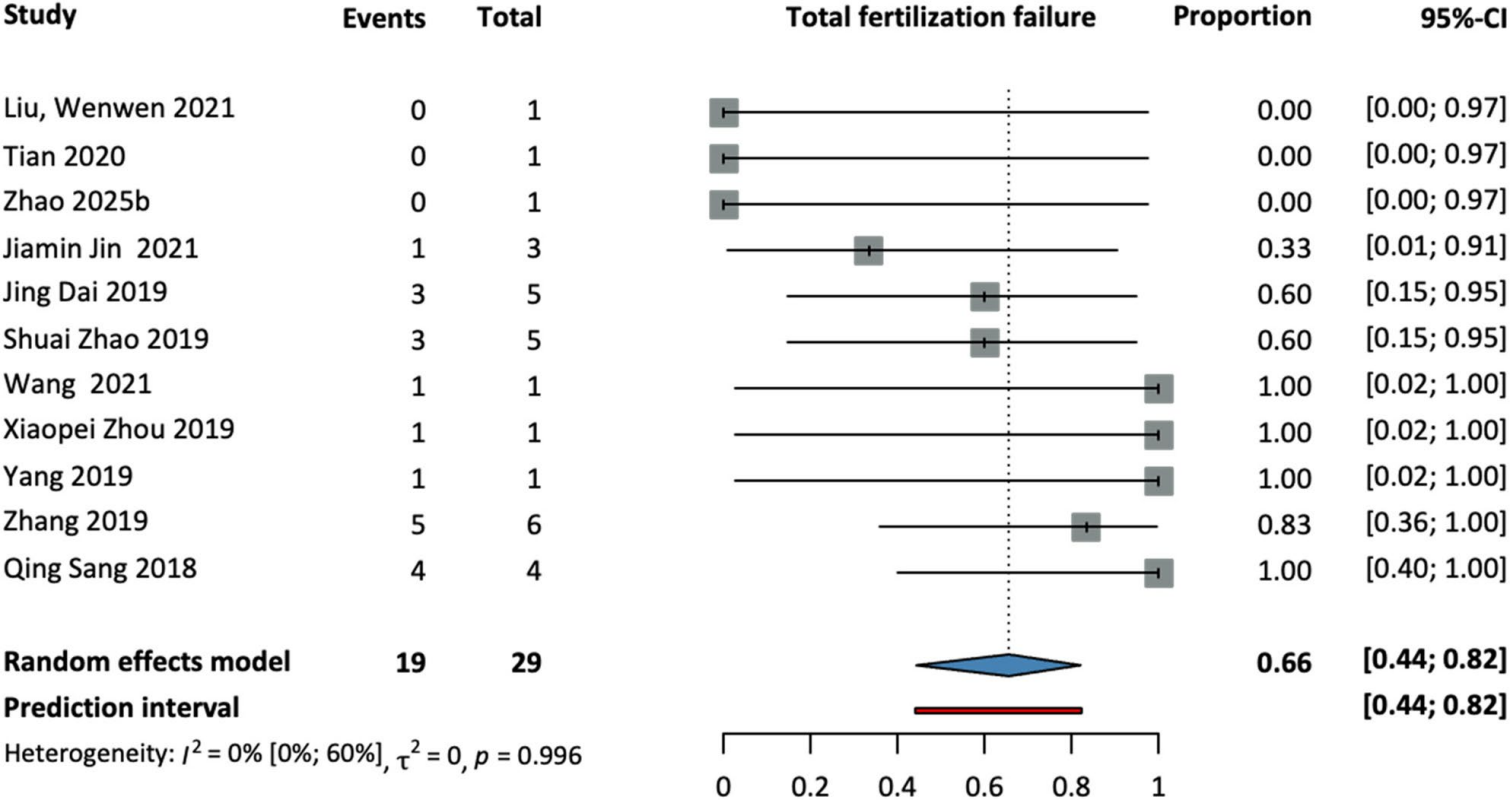
Forest plot on the proportion of TFF in patients with WEE2 mutation. Total: total number of patients. Events: TFF events

Figure 3 shows the effect of the WEE2 mutation on the TFF rates. A total of 11 studies were found, and WEE2 mutations were detected in 29 patients. Of these 29 patients, 19 experienced.

TFF, corresponding to a proportion of 66% (95% CI: 44–82%). This represents one of the OGMs associated with the highest rates of TFF.

All other plots showing the effects of other OGMs on TFF rates can be found in the Supplementary Material, *Figure S1*.

Finally, *Figures S2 and S3* depict TFF rates in the control indirect comparator group. *Figure S2* presents TFF data by patient, whereas *Figure S3* shows TFF data by cycle number. In the indirect comparator group, TFF rate was 9% (95% CI: 6–14%).

### Secondary outcome: FR

We included 64 articles in the oocyte gene mutation group, and 12 articles in the control indirect comparator group. We were unable to include all articles used for TFF in FR due to missing data.

A total of 7,314 retrieved oocytes were collected in the exposure group, of which 1,904 were fertilized. Certain OGMs, particularly WEE2 and PATL2 were associated with lower FR. Patients with WEE2 mutations had a FR of 3% (95% CI: 1–10%), and those with PATL2 mutations had an FR of 2% (95% CI: 0–10%).

**Fig. 4:**
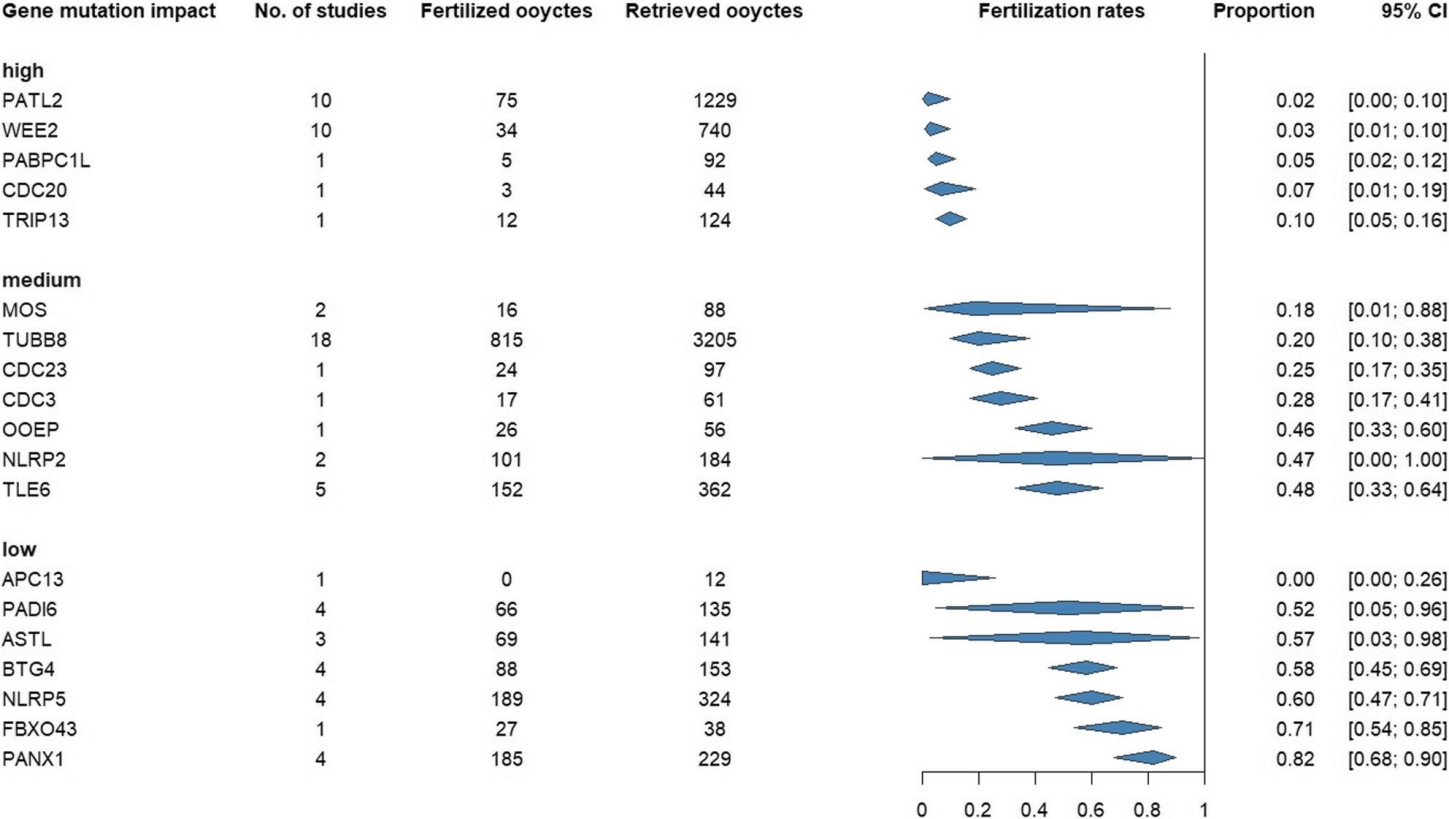
Summary plot showing the pooled fertilization rates in oocyte based aspect for different types OGMs grouped by the magnitude of impact.

Figure 4 presents a summary of the fertilization rates in the mutated population. The figure illustrates different types of oocyte mutations and their magnitude of impact on fertilization rates, showing the variety of effects that different types of mutations can have. The magnitude of impacts is grouped into high (between 0 and 10%), medium (between 10 and 50%), and low (50% and over). The high magnitude of impact reflects the OGMs’ ability to interfere with the fertilization process and to exert a restrictive effect on fertilization.

*Figure S4*, included in the Supplementary Material, shows the FR of the control indirect comparator group. This plot indicates the pooled proportion, which is 67% (95% CI: 62–71%). The summary forest plot in *Figure S5* shows the FR for each mutation separately. The highest FR was observed in the PANX1 mutation at 82% (95% CI: 68–90%), whereas the lowest was observed in PATL2 at 2% (95% CI: 0–10%).

### Risk of bias assessment

The results of the risk of bias assessment are presented in *Tables S2*, *S3*, and *S4*. Risk of bias was low for the majority of the included articles, as fertilization data were reported clearly and the infertility of women was clearly explained in each article.

### Heterogeneity and publication bias

When investigating heterogeneity, we considered three major aspects. First, we kept in mind that heterogeneity assessment is limited by the small number of studies are included in the meta-analyis. Second, the vast majority of the studies had very low sample sizes which inevitably led to wide confidence intervals. Third, when meta-analyzing proportions, the I^2^ statistic is reported on a logarithmic scale, whereas effect sizes are displayed on the regular scale for easier comprehension. So, we concluded that the I^2^ values have limited informative value in our study, therefore, we relied on visual inspection of the forest plots, focusing on the range of effect sizes across the individual studies and the overlap in the confidence intervals, which can be translated into clinical heterogeneity. In the forest plot showing the TFF rates in patients with WEE2 mutation (Fig. 3), although the I^2^ value is low there are considerable differences in individual effect sizes primarily due to sparse data and small patient numbers.

TFF rates and FR in the indirect comparator studies (*Figures S2* and *S4)* demonstrate substantial heterogeneity. This can be attributed to differences between the populations studied, stemming from diverse infertility backgrounds; which influence their assisted reproduction results. Due to the lack of data we didn’t investigate the heterogeneity further e.g. through subgroup analyses.

Our meta-analysis consisted only of single-arm studies, which means no significance testing was carried out and entails that publication bias in its most traditional form cannot be assessed. However, any overly influential effect of studies with small patient numbers and large effect sizes (small study bias) can still be present in single-arm meta-analyses. Regarding studies on patients with OGMs however, all studies involved minimal patient numbers, which limits the assessment of small-study effects. Furthermore, effect sizes in studies with zero event counts are continuity-corrected when visualized on funnel-plots (see *Figure S6* for the funnel plot and Peters’test p-value of the meta-analysis of TFF in patients with WEE2 mutations). Therefore, we decided not to draw any conclusions about publication bias for outcomes in the population with OGMs.

In the indirect comparator group, the study and patient numbers were considerably higher. *Figures S6* and *S7* show the funnel plots along with Peter’s test p-values for the TFF and fertilization rates in the non-genotyped population, respectively. The funnel plot for the FR in the non-genotyped population can be seen in *Figure S8*. Neither visually nor statistically was any asymmetry detected for these outcomes.

## Discussion

Our study demonstrates differences in assisted reproduction outcomes between patients with OGMs and non-genotyped patients (indirect comparators).

We found that some mutations such as NLRP2, NLRP5, and PANX1 showed no effect on TFF rates. Even though we did not analyze any events beyond TFF, the literature indicates that these mutations negatively influence the stages of blastocyst and embryo development [10]. Embryos derived from oocytes carrying these mutations tend to arrest at an early stage of development [29].

However, we observed some mutations, such as WEE2 and PATL2, with TFF rates reaching as high as 67%. The FR associated with these mutations tend to be low.

To our knowledge, there is a lack of comprehensive reviews in the literature assessing the effects of OGMs on fertilization outcomes such as TFF and FR in assisted reproduction. Previous reviews provide insights into how OGMs influence the phases of oocyte maturation and the resulting pathway disturbances that contribute to female infertility [10, 29–31]. Several mutations are associated with oocyte maturation defects, such as WEE2, PATL2, and TUBB8. WEE2 mutation interferes with the meiosis-regulating kinase in the oocyte, which is involved in oocyte activation [29]. PATL2 mutations disrupt maternal mRNA regulation, leading to oocyte maturation arrest and severely impaired fertilization outcomes [29]. TUBB8 mutations disrupt spindle assembly and chromosome alignment, leading to oocyte maturation arrest and compromised fertilization competence [29].

Attention has been paid to gene-based experimental strategies to improve the success of assisted reproduction. Sang et al. [31] describe a proof-of-concept approach in which WEE2 cRNA was injected into mutated oocytes, leading to successful fertilization and the development of morphologically normal blastocysts in an experimental setting. While these findings demonstrate the potential of cRNA supplementation to rescue oocyte activation defects, this approach remains investigational, and its safety, efficacy, and feasibility for clinical application have yet to be established.

In addition to addressing the challenges posed by OGM, it can be helpful to test women diagnosed with unexplained infertility or women with repeated unsuccessful cycles, for genetic mutations to better understand the underlying causes of infertility [32]. Knowledge of etiology and taking appropriate steps accordingly leads to more efficient and personalized ART [33, 34]. According to current guidelines, genetic testing for OGMs is not routinely considered [35]. However, our findings indicate that performing genetic testing may be beneficial for those with unexplained infertility and for women who have had multiple unsuccessful treatment attempts. This change could help prevent patients from undergoing unnecessary and ineffective interventions.

### Strengths and limitations

Our research has both strengths and limitations. A key strength is the use of a rigorous methodology, which ensured systematic extraction and analysis of data. While over 100 articles were included, providing context for interpretation, many studies involved small and dispersed populations, which limits the robustness of the conclusions.

A significant limitation of our study was the reliance on indirect assessment, mainly due to the scarcity of comparative studies. Much of the available evidence came from case reports and small case series rather than well-designed comparative studies. In articles examining the exposure group, all participants were screened for OGM; however, outcome data were reported only for individuals who tested positive. This selective reporting limited the ability to perform direct comparisons and may have affected the interpretation of the findings. In this context, we referred to studies that did not incorporate genetic testing and therefore primarily reported assisted reproduction outcomes in populations without known oocyte gene mutations, reflecting the low prevalence of such mutations. As a result, the non-genotyped arm represents an indirect comparison group, a design that may introduce selection bias and residual confounding, particularly given the heterogeneity of ART indications and treatment protocols across control studies. The articles in the exposure group primarily consist of case reports and small case series, while those in the indirect comparator group are mainly made up of cohort studies and randomized controlled trials. We recognize that the differences in the evidence base between these two sets of articles limit causal inference and their comparability. Last but not least, the majority of studies investigating exposure groups were conducted in China, which may limit the generalizability of the findings. However, the definition of TFF is universal, the fertilization techniques being used in the setting of ART are quite similar and protocoled which enables the interpretation of the results in a more global framework.

### Implications for practice and research

Implementing scientific findings in practice is crucial [36, 37]. TFF during ART is one of the most frustrating phenomena for both the patient and the physician. Therefore, with this study we would like to highlight that in some rare cases OGMs can be the cause when oocytes aren’t getting fertilized at all. We believe that offering genetic testing for women with unexplained infertility or repeated unsuccessful assisted reproduction cycles could be beneficial to diagnose these rare cases of infertility and help them choose an appropriate treatment option in the future. Although the prevalence of OGM is low, these mutations may have negative impacts on fertility. Understanding the underlying cause of female infertility and communicating this information can play an important role. Further studies are needed to directly compare the fertilization and TFF rates between OGM carriers and non-carriers. This will help determine which group of patients would benefit from genetic testing for OGMs and when exactly would be the appropriate time to test them during their infertility work up.

Further research on the OGMs should be conducted globally to eliminate limitations arising from results based on a restricted population. Experimental approaches on cRNA injection should be further researched and add more knowledge to the field. Research should also be expanded to gene editing methods that could increase success rates in assisted reproduction for women who are carriers of OGM.

## Conclusion

Our findings show that women carrying certain types of OGM exhibit higher TFF and lower FR, while these rates show an opposite pattern in a non-genotyped population. TFF during ART is a frustrating outcome for both patients and clinicians, and in rare cases targeted genetic testing for OGMs- that we highlighted in our study - can decrease the psychological burden of multiple unsuccessful treatments.

## Supplementary Information

Supplementary Material 1.

## Data Availability

No datasets were generated or analysed during the current study.
