# Supplementary material for: Oocyte gene mutations increase rates of total fertilization failure: a systematic review and meta-analysis
Source: Reprod Biol Endocrinol. 2026 Apr 18;24:56. doi: 10.1186/s12958-026-01546-9 (PMC13227840; doi:10.1186/s12958-026-01546-9)
Supplement: Supplementary file 1 — Supplementary Material 1. [file 12958_2026_1546_MOESM1_ESM.docx]

**Oocyte gene mutations increase the rates of total fertilization failure and result in lower fertilization rates: A Systematic Review and Meta-analysis**

**Authors**

Begüm Kepkep^1^, Máte Szabolcs Botos^1,7^, İpek Yazıcı, Lőrinc Frivaldszky^1,3,6^, Anett Rancz^1^, Péter Hegyi^1,2,4,5^, Nándor Ács^1,3^, Zsolt Melczer ^1,3^, Boglárka Szentes^1^, Miklós Sipos^1,3^

**Affiliations:**

1. Centre for Translational Medicine, Semmelweis University, Budapest, Hungary
2. Institute of Pancreatic Diseases, Semmelweis University, Budapest, Hungary
3. Department of Obstetrics and Gynecology, Semmelweis University, Budapest, Hungary
4. Translational Pancreatology Research Group, Interdisciplinary Centre of Excellence for Research Development and Innovation University of Szeged, Szeged, Hungary
5. Institute for Translational Medicine, Medical School, University of Pécs, Pécs, Hungary
6. MRE Bethesda Children’s Hospital, Budapest, Hungary
7. Fejér County Szent György University Teaching Hospital, Székesfehérvár, Hungary

**Corresponding author**

Miklos Sipos MD, PhD

Postal address: Gyali ut 17-19. Building 30., 1097, Budapest, Hungary

Tel.: +36 30954 5908

E-mail address: [sipos.miklos@semmelweis.hu](mailto:sipos.miklos@semmelweis.hu)

**Supplementary Materials**

**Figures and tables in supplementary materials**

**Appendix S1: Search Strategy**

**Table S1: Baseline Characteristics Table**

**Table S2: Risk of Bias Assessment for Case Reports**

**Table S3: Risk of Bias Assessment for Case Series**

**Table S4: Risk of Bias Assessment for Prevalence** (JBI Checklist for prevalence was used to assess the risk of bias in the control group articles)

**Figure S1: Total Fertilization Failure rates in patients with different oocyte gene mutations (based on patients)**

**Figure S2: Total Fertilization Failure rates in the indirect comparator group (based on patients)**

**Figure S3: Total Fertilization Failure rates in the indirect comparator group (based on cycles)**

**Figure S4: Fertilization rates in the indirect comparator group**

**Figure S5: Fertilization rates in patients with different oocyte gene mutations**

**Figure S6: Funnel plot of TFF rates in patients with WEE2 mutations**

**Figure S7: Funnel plot of TFF rates in patients with no genetic testing**

**Figure S8: Funnel plot of fertilization rates in patients with no genetic testing**

**References**

**Appendix S1: Search Strategy**

The following search key was used for PubMed with the so called “[tiab:~5]” function: ((("in vitro" OR "in-vitro") AND "fertilization") OR "IVF" OR "intracytoplasmic sperm injection" OR "ICSI" OR ("oocyte" AND matur*) OR ("assisted" AND reprod*) OR "ART" OR (("woman" OR "women" OR "female" OR "females") AND (infert* OR subfert* OR sub-fert* OR steril*))) AND ("fertilization failure"[tiab:~5] OR "FF" OR "fertility failure"[tiab:~5] OR "poor fertility"[tiab:~5] OR "poor fertilization"[tiab:~5]).

Moreover we used the following search key for EMBASE and Cochrane Library with the “NEAR/5” function: ('in vitro' AND fertilization OR 'ivf' OR 'intracytoplasmic sperm injection' OR 'icsi' OR ('oocyte' AND matur*) OR ('assisted' AND reprod*) OR 'art' OR (('woman' OR 'women' OR 'female' OR 'females') AND (infert* OR subfert* OR 'sub fert*' OR steril*))) AND ((fertil* NEAR/5 fail*) OR 'ff' OR ('poor' NEAR/5 fert*))

**Table S1: Baseline Characteristic Table**

| **Author (year)** | **Study Site** | **Exposure** | **Outcomes** | **Number of patients with the given exposure** | **Mean age (standard deviation) of women** |
| --- | --- | --- | --- | --- | --- |
| Chen et.al_2016 [1] | China | TUBB8 | TFF, Fertilization Rates | 10 | 32,4 ±  5 |
| Jin et.al_2021 [2] | China | WEE2 | TFF, Fertilization Rates | 3 | 29,3 ± 0,6 |
| Dai et.al_2019 [3] | China | WEE2 | TFF, Fertilization Rates | 5 | 29,4 ±  2,2 |
| Huang et.al_2018 [4] | China | PATL2 | TFF, Fertilization Rates | 4 | 31,8 ± 3,4 |
| Maddirevula et.al_2017 [5] | Saudi Arabia | PATL2 | TFF, Fertilization Rates | 3 | NA |
| Zhao et.al_2019 [6] | China | WEE2 | TFF, Fertilization Rates | 5 | 30,2 ±  5,7 |
| Sang et.al_2018 [7] | China | WEE2 | TFF, Fertilization Rates | 4 | 31,8 ± 4,6 |
| Zhou et.al_2019 [8] | China | WEE2 | TFF, Fertilization Rates | 1 | 27 |
| Liu et.al_2020 [9] | China | PATL2 | TFF, Fertilization Rates | 3 | 35,3 ± 4,7 |
| Chen et.al_2017 [10] | China | PATL2 | TFF, Fertilization Rates | 5 | 31,4 ±  1,1 |
| Lin et.al_2023 [11] | China | TUBB8 | TFF, Fertilization Rates | 15 | 28,9 ± 2,7 |
| Zhao et.al_2019 [12] | China | TUBB8 | TFF, Fertilization Rates | 37 | 31,1 ±  3,7 |
| Hu et.al_2023 [13] | China | TUBB8 | TFF, Fertilization Rates | 9 | 31 ±  3,1 |
| Cao et.al_2021 [14] | China | TUBB8 | TFF, Fertilization Rates | 2 | 31,5 ± 2,1 |
| Chen et.al_2019 [15] | China | TUBB8 | TFF, Fertilization Rates | 28 | 30,8 ±  3,6 |
| Akbari et.al_2022 [16] | Iran | TLE6 | TFF, Fertilization Rates | 3 | 41,7 ±  12,7 |
| Cao et.al_2021 [17] | China | PATL2 | TFF, Fertilization Rates | 6 | 28,7 ± 2,5 |
| Cao et.al_2023 [18] | China | PADI6 | TFF, Fertilization Rates | 1 | 28 |
| Chai et.al_2024 [19] | China | NLRP2 | TFF, Fertilization Rates | 1 | 29 |
| Dong et.al_2021 [20] | China | MEI1 | TFF, Fertilization Rates | 7 | 35,3 ±  3,1 |
| Dou et.al_2023 [21] | China | TUBB8 | TFF, Fertilization Rates | 2 | 28 ± 1,4 |
| Fan et.al_2023 [22] | China | CDC3 | TFF, Fertilization Rates | 3 | 27 ±  3,5 |
| Hu et.al_2024 [23] | China | PATL2 | TFF, Fertilization Rates | 3 | 31,7 ±  3,1 |
| Huang et.al_2022 [24] | China | NLRP5 and PATL2 | TFF, Fertilization Rates | 2 | 37,5 ±  4,9 |
| Jia et.al_2020 [25] | China | TUBB8 | TFF, Fertilization Rates | 1 | 38 |
| Jiao et.al_2022 [26] | China | MOS | TFF, Fertilization Rates | 1 | 25 |
| Lanuza-Lopez et.al_2020 [27] | Mexico | TUBB8 | TFF, Fertilization Rates | 3 | 33,7 ±  3,1 |
| Li et.al_2024 [28] | China | NLRP5 and TLE6 | TFF, Fertilization Rates | 2 | 32,5 ±  0,7 |
| Lin et.al_2020 [29] | China | TLE6 | TFF, Fertilization Rates | 3 | 31,7 ±  4,9 |
| Liu, Ruyi et.al_2021 [30] | China | BTG4 | TFF, Fertilization Rates | 1 | 35 |
| Liu, Juan et.al_2021 [31] | China | PADI6 and TLE6 | TFF, Fertilization Rates | 2 | 30 ±  2,8 |
| Liu, Zhenxing et.al_2021 [32] | China | TUBB8 | TFF, Fertilization Rates | 2 | 30 ± 2,8 |
| Liu, Wenwen et.al_2021[33] | China | WEE2 | TFF, Fertilization Rates | 1 | 37 |
| Maddirevula et.al_2021 [34] | Saudi Arabia | ASTL | TFF, Fertilization Rates | 1 | 28 |
| Mao et.L_2021 [35] | China | TLE6 | TFF, Fertilization Rates | 1 | 32 |
| Mu et.al_2019 [36] | China | NLRP5 and NLRP2 | TFF, Fertilization Rates | 8 | 28,3 ±  2,7 |
| Okutman et.al_2024 [37] | Belgium, Turkey, France | PABPC1L/EPAB | TFF, Fertilization Rates | 1 | NA |
| Tong et.al_2022[38] | China | OOEP and NLRP5 | TFF, Fertilization Rates | 5 | 31,9 ±  7,7 |
| Wang et.al_2021 [39] | China | WEE2 | TFF, Fertilization Rates | 1 | 36 |
| Wang et.al_2021 [40] | China | FBXO43 | TFF, Fertilization Rates | 3 | 31 ± 1,4 |
| Wang et.al_2021 [41] | China | PANX1 | TFF, Fertilization Rates | 2 | 32 ± 5,7 |
| Wang et.al_2022 [42] | China | PADI6 | TFF, Fertilization Rates | 1 | 34 |
| Wang et.al_2023 [43] | China | BTG4 | TFF, Fertilization Rates | 1 | 31 |
| Wang et.al_2023 [44] | China | PABPC1L | TFF, Fertilization Rates | 5 | 29,8 ±  3,3 |
| Weiner et.al_2022 [45] | USA | WEE2 | TFF, Fertilization Rates | 1 | 25 |
| Wu et.al_2022 [46] | China | PANX1 | TFF, Fertilization Rates | 1 | 33 |
| Wu et.al_2023 [47] | China | ASTL | TFF, Fertilization Rates | 1 | 27 |
| Xu et.al_2020 [48] | China | NLRP5 | TFF, Fertilization Rates | 1 | 28 |
| Yang et.al_2019 [49] | China | WEE2 | TFF, Fertilization Rates | 1 | 27 |
| Yang et.al_2021 [50] | China | TUBB8 | TFF, Fertilization Rates | 36 | NA |
| Yao et.al_2022 [51] | China | TUBB8 | TFF, Fertilization Rates | 5 | 31 ±  2,5 |
| Ye et.al_2024 [52] | China | PATL2 | TFF, Fertilization Rates | 15 | 31,5 ±  3 |
| Yu et.al_2023 [53] | China | TUBB8 | TFF, Fertilization Rates | 5 | 30 ±  3,9 |
| Zeng et.al_2023 [54] | China | ASTL | TFF, Fertilization Rates | 4 | 31,5 ±  4,9 |
| Zhang et.al_2019 [55] | China | WEE2 | TFF, Fertilization Rates | 6 | 33,3 ± 4,3 |
| Zhang et.al_2020 [56] | China | TRIP13 | TFF, Fertilization Rates | 4 | 32 ± 5,3 |
| Zhang et.al_2021 [57] | China | TLE6 | TFF, Fertilization Rates | 4 | 33,5 ± 2,4 |
| Zhang et.al_2022 [58] | China | MOS | TFF, Fertilization Rates | 3 | 28,3 ±  0,6 |
| Zhang et.al_2023 [59] | China | TUBB8 | TFF, Fertilization Rates | 2 | 33,5 ± 6,4 |
| Zhang et.al_2024 [60] | China | TUBB8 | TFF, Fertilization Rates | 1 | NA |
| Zhao et.al_2021 [61] | China | CDC20 | TFF, Fertilization Rates | 3 | 34 ±  2,6 |
| Zheng et.al_2020 [62] | China | BTG4 | TFF, Fertilization Rates | 4 | 29 ±  3,3 |
| Zhou et.al_2024 [63] | China | PANX1 | TFF, Fertilization Rates | 2 | NA |
| Zhou et.al_2024 [64] | China | PADI6 | TFF, Fertilization Rates | 2 | 32 ± 2,8 |
| Zhou et.al_2023 [65] | China | PANX1 | TFF, Fertilization Rates | 1 | 33 |
| Zhu et.al_2022 [66] | China | TUBB8 and PATL2 | TFF, Fertilization Rates | 7 | 32,6 ±  3,8 |
| Liu et.al_2024 [67] | China | PATL2 and WEE2 | TFF, Fertilization Rates | 2 | 32 |
| Sha et.al_2021 [68] | China | TUBB8 | TFF, Fertilization Rates | 3 | 30,7 ±  3,1 |
| Tian et.al_2020 [69] | China | WEE2 | TFF, Fertilization Rates | 1 | 36 |
| Wu et.al_2019 [70] | China | PATL2 | TFF, Fertilization Rates | 7 | 29,7 ±  5 |
| Yuan et.al_2018 [71] | China | TUBB8 | TFF, Fertilization Rates | 1 | 34 |
| Aboulghar et.al_1999 [72] | Egypt | - | TFF, Fertilization Rates | 485 | 32 ± 4,5 |
| Benadiva et.al_1999 [73] | USA | - | TFF | 25 | NA |
| Biliangady et.al_2019 [74] | India | - | TFF | 350 | NA |
| Briton-Jones et.al_2009 [75] | USA | - | TFF, Fertilization Rates | 306 | NA |
| Bukulmez et.al_ 2000 [76] | Turkey | - | TFF | 76 | NA |
| Chiamchanya et.al_2008 [77] | Thailand | - | TFF, Fertilization Rates | 36 | 33,25 + 4,22 |
| Dang et.al_2021 [78] | Vietnam, Australia, UK | - | TFF | 1064 | NA |
| De Souza et.al_2023 [79] | Brazil | - | TFF | 629 | NA |
| Ebner et.al_2011 [80] | Austria | - | TFF, Fertilization Rates | 50 | 32,9 ± 4,1 |
| Foong et.al_2006 [81] | Canada | - | TFF | 60 | NA |
| Gennarelli et.al_2019 [82] | Italy | - | TFF | 604 | NA |
| Gil Raga et.al_2005 [83] | NA | - | TFF, Fertilization Rates | NA | NA |
| Hershlag et.al_2001 [84] | USA | - | TFF | 60 | NA |
| Hwang et.al_2005 [85] | Taiwan | - | TFF, Fertilization Rates | 60 | 32,5  ±  2,3 |
| Jaroudi et.al_2003 [86] | Saudi Arabia | - | TFF, Fertilization Rates | 125 | NA |
| Johnson et.al_2015 [87] | USA | - | TFF | NA | NA |
| Kim et.al_2013 [88] | South Korea | - | TFF, Fertilization Rates | 217 | NA |
| Kim et.al_2007 [89] | USA | - | TFF | 486 | NA |
| Majumdar et.al_2010 [90] | NA | - | TFF | 116 | NA |
| Moreno et.al_1998 [91] | Spain | - | TFF | 96 | NA |
| Nachef et.al_2009 [92] | NA | - | TFF | 661 | 35,9 |
| Ou, Yu-Che et.al_2010 [93] | Taiwan | - | TFF | NA | NA |
| Ruiz et.al_1997 [94] | Spain | - | TFF, Fertilization Rates | 140 | 31,9 ± 0,4 |
| Takeuchi et.al_2000 [95] | Japan | - | TFF, Fertilization Rates | 128 | NA |
| Tannus et.al_2017 [96] | Canada | - | TFF | 745 | NA |
| Tondo et.al_2023 [97] | Italy | - | TFF | 439 | NA |
| Vigano et.al_2023 [98] | Italy, Brazil | - | TFF | 157 | NA |
| Wyns et.al_2004 [99] | Belgium | - | TFF | 30 | NA |
| Youssef et.al_2009 [100] | Egypt | - | TFF, Fertilization Rates | 112 | NA |
| Li, Zhiling et.al_2004 [101] | China | - | TFF | 11 | 33,9 ± 5,3 |
| Zhu et.al_2024 [102] | China | - | TFF, Fertilization Rates | 547 | NA |

| Cui et.al 2025 [103] |  | CDC23 and APC13 | TFF, Fertilization Rates | 3 | 28 ± 2,3 |
| --- | --- | --- | --- | --- | --- |
| Li, Hongyan et.al 2025 [104] |  | PATL2 | TFF, Fertilization Rates | 1 |  |
| Zhao, Haijing et.al 2025 [105] |  | WEE2, PATL2 and TUBB8 | TFF, Fertilization Rates | 5 |  |

**Table S2: Risk of Bias Assessment Tables for Case Reports**

|  | Were patient’s demographic characteristics clearly described? | Was the patient’s history clearly described and presented as a timeline? | Was the current clinical condition of the patient on presentation clearly described? | Were diagnostic tests or assessment methods and the results clearly described? | Was the intervention(s) or treatment procedure(s) clearly described? | Was the post-intervention clinical condition clearly described? | Were adverse events (harms) or unanticipated events identified and described? | Does the case report provide takeaway lessons? | Overall |
| --- | --- | --- | --- | --- | --- | --- | --- | --- | --- |
| Cao et.al_2023 | Yes | Yes | Yes | Yes | Not applicable | Not applicable | Not applicable | Yes | Include |
| Chai et.al_2024 | Yes | Yes | Yes | Yes | Not applicable | Not applicable | Not applicable | Yes | Include |
| Jia et.al_2020 | Yes | Yes | No | No | Not applicable | Not applicable | Not applicable | Unclear | Include |
| Jiao et.al_2022 | Yes | Yes | Yes | Yes | Not applicable | Not applicable | Not applicable | Yes | Include |
| Liu et.al_2021 | Yes | Yes | Yes | Yes | Not applicable | Not applicable | Not applicable | Yes | Include |
| Liu et.al_2021 | Yes | Yes | No | No | Not applicable | Not applicable | Not applicable | Yes | Include |
| Mao et.al_2021 | Yes | Yes | Yes | Yes | Not applicable | Not applicable | Not applicable | Yes | Include |
| Tian et.al_2020 | Yes | No | Yes | No | Not applicable | Not applicable | Not applicable | Yes | Include |
| Wang et.al_2022 | Yes | Yes | Yes | Yes | Not applicable | Not applicable | Not applicable | Yes | Include |
| Wang et.al_2023 | Yes | Yes | Yes | Yes | Not applicable | Not applicable | Not applicable | Yes | Include |
| Weiner et.al_2022 | Yes | Yes | Yes | Yes | Not applicable | Not applicable | Not applicable | Yes | Include |
| Xu et.al_2020 | Yes | Yes | No | No | Not applicable | Not applicable | Not applicable | Yes | Include |
| Yang et.al_2019 | Yes | Yes | Yes | No | Not applicable | Not applicable | Not applicable | Yes | Include |
| Yuan et.al_2018 | Yes | Yes | Yes | Yes | Not applicable | Not applicable | Not applicable | Yes | Include |
| Zhang et.al_2024 | Yes | Yes | Yes | Yes | Not applicable | Not applicable | Not applicable | Yes | Include |

**Table S3: Risk of Bias Assessment for Case Series**

|  | Were there clear criteria for inclusion in the case series? | Was the condition measured in a standard, reliable way for all participants included in the case series? | Were valid methods used for identification of the condition for all participants included in the case series? | Did the case series have consecutive inclusion of participants? | Did the case series have complete inclusion of participants? | Was there clear reporting of the demographics of the participants in the study? | Was there clear reporting of clinical information of the participants? | Were the outcomes or follow up results of cases clearly reported? | Was there clear reporting of the presenting site(s)/clinic(s) demographic information? | Was statistical analysis appropriate? | Overall |
| --- | --- | --- | --- | --- | --- | --- | --- | --- | --- | --- | --- |
| Chen et.al_2016 | Yes | Yes | Yes | Yes | Yes | Yes | Yes | Yes | Yes | Not applicable | Include |
| Jin et.al_2021 | Yes | Yes | Yes | Yes | No | Yes | Yes | Yes | Yes | Not applicable | Include |
| Dai et.al_2019 | Yes | Yes | Yes | Yes | No | Yes | No | Yes | Yes | Not applicable | Include |
| Huang et.al_2018 | Yes | Yes | Yes | Yes | No | Yes | Yes | Yes | Yes | Not applicable | Include |
| Zhao et.al_2019 | Yes | Yes | Yes | Yes | No | Yes | No | Yes | Yes | Not applicable | Include |
| Sang et.al_2018 | Yes | Yes | Yes | Yes | Yes | Yes | Yes | Yes | Yes | Not applicable | Include |
| Zhou et.al_2019 | Yes | Yes | Yes | Yes | No | Yes | Yes | Yes | Yes | Not applicable | Include |
| Liu et.al_ 2020 | Yes | Yes | Yes | Yes | No | Yes | Yes | Yes | Yes | Not applicable | Include |
| Chen et.al_2017 | Yes | Yes | Yes | Yes | No | Yes | No | Yes | Yes | Not applicable | Include |
| Lin et.al_2023 | Yes | Yes | Yes | Yes | No | Yes | Yes | Yes | Yes | Not applicable | Include |
| Zhao et.al_2020 | Yes | Yes | Yes | Yes | No | Yes | No | Yes | Yes | Not applicable | Include |
| Hu et.al_2023 | Yes | Yes | Yes | Yes | No | Yes | No | Yes | Yes | Not applicable | Include |
| Cao et.al_2021 | Yes | Yes | Yes | Yes | Yes | Yes | Yes | Yes | Yes | Not applicable | Include |
| Chen et.al_2019 | Yes | Yes | Yes | Yes | No | Yes | Yes | Yes | Yes | Not applicable | Include |
| Akbari et.al_2022 | Yes | Yes | Yes | Yes | Unclear | Yes | Yes | Yes | Yes | Not applicable | Include |
| Cao et.al_2021 | Yes | Yes | Yes | Yes | Unclear | Yes | Yes | Yes | Yes | Not applicable | Include |
| Dong et.al_2021 | Yes | Yes | Yes | Yes | No | Yes | Yes | Yes | Yes | Not applicable | Include |
| Dou et.al_2023 | Yes | Yes | Yes | Yes | Unclear | Yes | Yes | Yes | Yes | Not applicable | Include |
| Fan et.al_2023 | Yes | Yes | Yes | Yes | Yes | Yes | Yes | Yes | Yes | Not applicable | Include |
| Hu et.al_2024 | Yes | Yes | Yes | Yes | Unclear | Yes | Yes | Yes | Yes | Not applicable | Include |
| Huang et.al_2022 | Yes | Yes | Yes | Yes | No | Yes | Yes | Yes | Yes | Not applicable | Include |
| Lanuza-Lopez et.al_2020 | Yes | Yes | Yes | Yes | Yes | Yes | Yes | Yes | Yes | Not applicable | Include |
| Li et.al_2024 | Yes | Yes | Yes | Yes | Yes | Yes | Yes | Yes | Yes | Not applicable | Include |
| Lin et.al_2020 | Yes | Yes | Yes | Yes | No | Yes | Yes | Yes | Yes | Not applicable | Include |
| Liu et.al_2021 | Yes | Yes | Yes | Yes | Unclear | Yes | Yes | Yes | Yes | Not applicable | Include |
| Liu et.al_2021 | Yes | Yes | Yes | Yes | Yes | Yes | Yes | Yes | Yes | Not applicable | Include |
| Liu et.al_2024 | Yes | Yes | Yes | Yes | Yes | Yes | Yes | Yes | Yes | Not applicable | Include |
| Maddirevula et.al_2021 | Yes | Yes | Yes | Yes | Unclear | Yes | Yes | Yes | Yes | Not applicable | Include |
| Mu et.al_2019 | Yes | Yes | Yes | Yes | Unclear | Yes | Yes | Yes | Yes | Not applicable | Include |
| Okutman et.al_2024 | Yes | Yes | Yes | Yes | No | Yes | Yes | Yes | Yes | Not applicable | Include |
| Tong et.al_2022 | Yes | Yes | Yes | Yes | No | Yes | Yes | Yes | Yes | Not applicable | Include |
| Wang et.al_2021 | Yes | Yes | Yes | Yes | No | Yes | Yes | Yes | Yes | Not applicable | Include |
| Wang et.al_2021 | Yes | Yes | Yes | Yes | No | Yes | Yes | Yes | Yes | Not applicable | Include |
| Wang et.al_2021 | Yes | Yes | Yes | Yes | Unclear | Yes | Yes | Yes | Yes | Not applicable | Include |
| Wang et.al_2023 | Yes | Yes | Yes | Yes | No | Yes | Yes | Yes | Yes | Not applicable | Include |
| Wu et.al_2019 | Yes | Yes | Yes | Yes | No | Yes | Yes | Yes | Yes | Not applicable | Include |
| Wu et.al_2022 | Yes | Yes | Yes | Yes | Unclear | Yes | Yes | Yes | Yes | Not applicable | Include |
| Wu et.al_2023 | Yes | Yes | Yes | Yes | Unclear | Yes | Yes | Yes | Yes | Not applicable | Include |
| Yao et.al_2022 | Yes | Yes | Yes | Yes | No | Yes | No | Yes | Yes | Not applicable | Include |
| Ye et.al_2024 | Yes | Yes | Yes | Yes | No | Yes | No | Yes | Yes | Not applicable | Include |
| Yu et.al_2023 | Yes | Yes | Yes | Yes | Yes | Yes | Yes | Yes | Yes | Not applicable | Include |
| Zeng et.al_2023 | Yes | Yes | Yes | Yes | Yes | Yes | Yes | Yes | Yes | Not applicable | Include |
| Zhang et.al_2019 | Yes | Yes | Yes | Yes | Yes | Yes | Yes | Yes | Yes | Not applicable | Include |
| Zhang et.al_2020 | Yes | Yes | Yes | Yes | No | Yes | Yes | Yes | Yes | Not applicable | Include |
| Zhang et.al_2021 | Yes | Yes | Yes | Yes | No | Yes | Yes | Yes | Yes | Not applicable | Include |
| Zhang et.al_2022 | Yes | Yes | Yes | Yes | Unclear | Yes | Yes | Yes | Yes | Not applicable | Include |
| Zhang et.al_2023 | Yes | Yes | Yes | Yes | Unclear | Yes | Yes | Yes | Yes | Not applicable | Include |
| Zhao et.al_2021 | Yes | Yes | Yes | Yes | No | Yes | Yes | Yes | Yes | Not applicable | Include |
| Zheng et.al_2020 | Yes | Yes | Yes | Yes | Unclear | Yes | Yes | Yes | Yes | Not applicable | Include |
| Zhou et.al_2024 | Yes | Yes | Yes | Yes | Unclear | Yes | Yes | Yes | Yes | Not applicable | Include |
| Zhou et.al_2024 | Yes | Yes | Yes | Yes | Unclear | Yes | Yes | Yes | Yes | Not applicable | Include |
| Zhu et.al_2022 | Yes | Yes | Yes | Yes | No | Yes | Yes | Yes | Yes | Not applicable | Include |

**Table S4: Risk of Bias Assessment for Prevalence**

|  | Was the sample frame appropriate to address the target population? | Were study participants sampled in an appropriate way? | Was the sample size adequate? | Were the study subjects and the setting described in detail? | Was the data analysis conducted with sufficient coverage of the identified sample? | Were valid methods used for the identification of the condition? | Was the condition measured in a standard, reliable way for all participants? | Was there appropriate statistical analysis? | Was the response rate adequate, and if not, was the low response rate managed appropriately? | Overall |
| --- | --- | --- | --- | --- | --- | --- | --- | --- | --- | --- |
| Aboulgar et.al_ 1999 | Yes | Yes | Yes | Yes | Yes | Unclear | Yes | Not appropriate | Not appropriate | Include |
| Benadiva et.al_ 1999 | Yes | Yes | No | Yes | Yes | Unclear | Yes | Not appropriate | Not appropriate | Include |
| Biliangady et.al_2019 | Yes | Yes | Yes | Yes | Yes | Unclear | Yes | Not appropriate | Not appropriate | Include |
| Briton-Jones et.al_2009 | Conference abstract | | | | | | | | | |
| Bukulmez et.al_2000 | Yes | Yes | No | Yes | Yes | Unclear | Yes | Not appropriate | Not appropriate | Include |
| Chiamchanya et.al_2008 | Yes | Yes | No | Yes | Yes | Unclear | Yes | Not appropriate | Not appropriate | Include |
| Dang et.al_2021 | Yes | Yes | Yes | Yes | Yes | Yes | Yes | Not appropriate | Not appropriate | Include |
| De Souza et.al_2023 | Yes | Yes | Yes | Yes | Yes | Yes | Yes | Not appropriate | Not appropriate | Include |
| Ebner et.al_2011 | Yes | Yes | No | Yes | Yes | Unclear | Yes | Not appropriate | Not appropriate | Include |
| Foong et.al_2006 | Yes | Yes | No | Yes | Yes | Unclear | Yes | Not appropriate | Not appropriate | Include |
| Gennarelli et.al_2019 | Yes | Yes | Yes | Yes | Yes | Yes | Yes | Not appropriate | Not appropriate | Include |
| Gil Raga et.al_2005 | Conference abstract | | | | | | | | | |
| Hershlag et.al_2001 | Yes | Yes | No | Yes | Yes | Unclear | Yes | Not appropriate | Not appropriate | Include |
| Hwang et.al_2005 | Yes | Yes | No | Yes | Yes | Unclear | Yes | Not appropriate | Not appropriate | Include |
| Jaroudi et.al_ 2003 | Yes | Yes | Yes | Yes | Yes | Unclear | Yes | Not appropriate | Not appropriate | Include |
| Johnson et.al_2015 | Conference abstract | | | | | | | | | |
| Kim et.al_ 2013 | Yes | Yes | Yes | Yes | Yes | Unclear | Yes | Not appropriate | Not appropriate | Include |
| Kim et.al_2007 | Yes | Yes | Yes | Yes | Yes | Unclear | Yes | Not appropriate | Not appropriate | Include |
| Majumdar et.al_ 2010 | Conference abstract | | | | | | | | | |
| Moreno et.al_1998 | Yes | Yes | No | Yes | Yes | Unclear | Yes | Not appropriate | Not appropriate | Include |
| Nachef et.al_2009 | Conference abstract | | | | | | | | | |
| Ou, Yu-Che et.al_2010 | Yes | Yes | Yes | Yes | Yes | Yes | Yes | Not appropriate | Not appropriate | Include |
| Ruiz et.al_1997 | Yes | Yes | No | Yes | Yes | Unclear | Yes | Not appropriate | Not appropriate | Include |
| Takeuchi et.al_2000 | Yes | Yes | Yes | Unclear | Yes | Unclear | Yes | Not appropriate | Not appropriate | Include |
| Tannus et.al_2017 | Yes | Yes | Yes | Yes | Yes | Yes | Yes | Not appropriate | Not appropriate | Include |
| Tondo et.al_2023 | Conference abstract | | | | | | | | | |
| Vigano et.al_2023 | Yes | Yes | Yes | Yes | Yes | Yes | Yes | Not appropriate | Not appropriate | Include |
| Wyns et.al_2004 | Yes | Yes | No | Yes | Yes | Unclear | Yes | Not appropriate | Not appropriate | Include |
| Youssef et.al_2009 | Conference abstract | | | | | | | | | |
| Zhilling et.al_ 2004 | Yes | Yes | No | Yes | Yes | Unclear | Yes | Not appropriate | Not appropriate | Include |
| Zhu et.al_2024 | Yes | Yes | Yes | Yes | Yes | Yes | Yes | Not appropriate | Not appropriate | Include |

**Figure S1. Total fertilization failure in patient with oocyte gene mutations (patient based)**

**
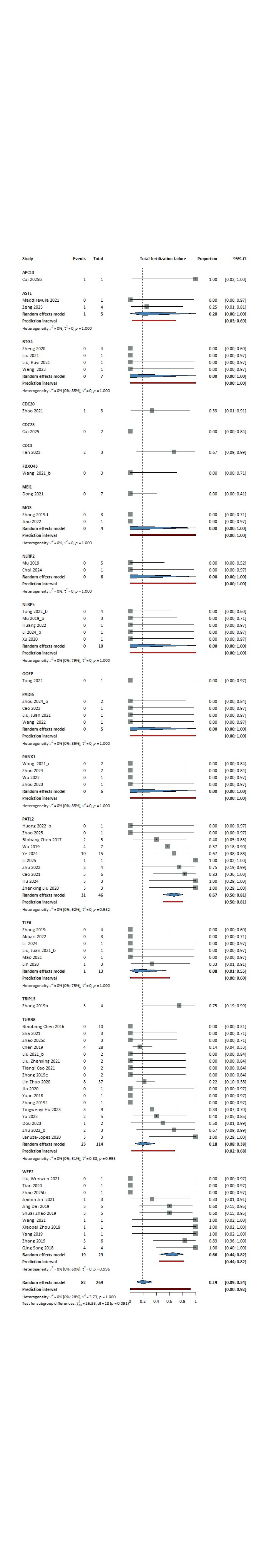
**

**Figure S2: Total Fertilization Failure rates in indirect comparator group patients (based on patients)**


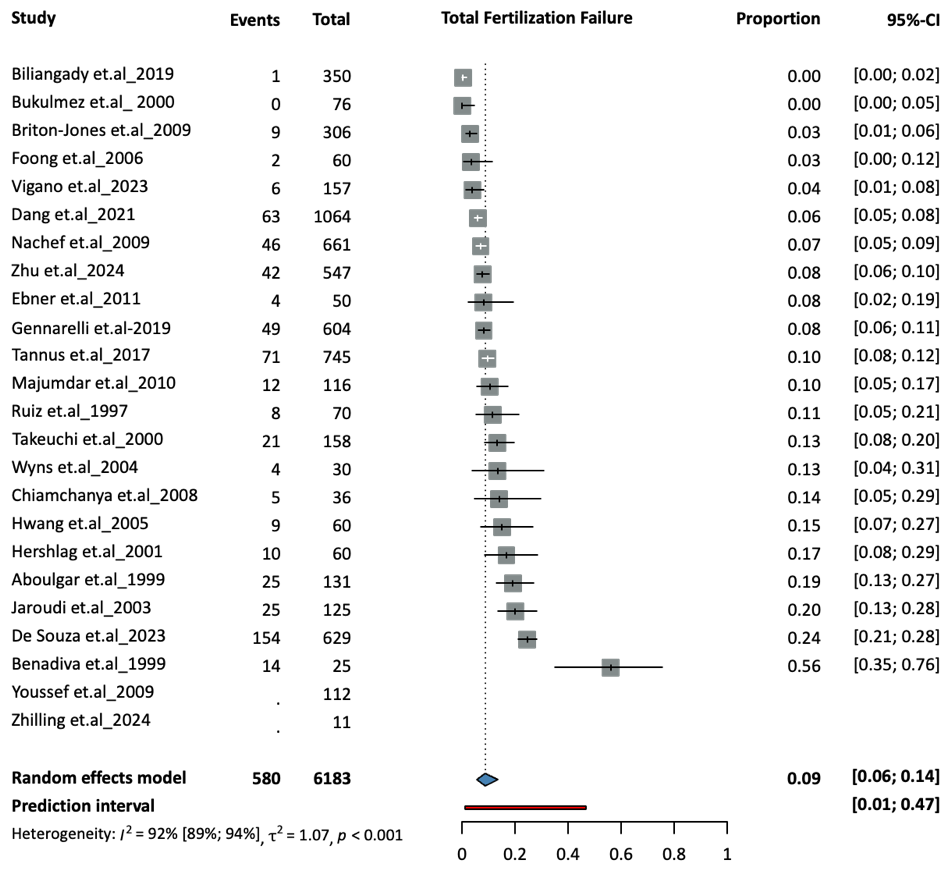


**Figure S3: Total Fertilization Failure rates in the indirect comparator group patients (based on cycles)**


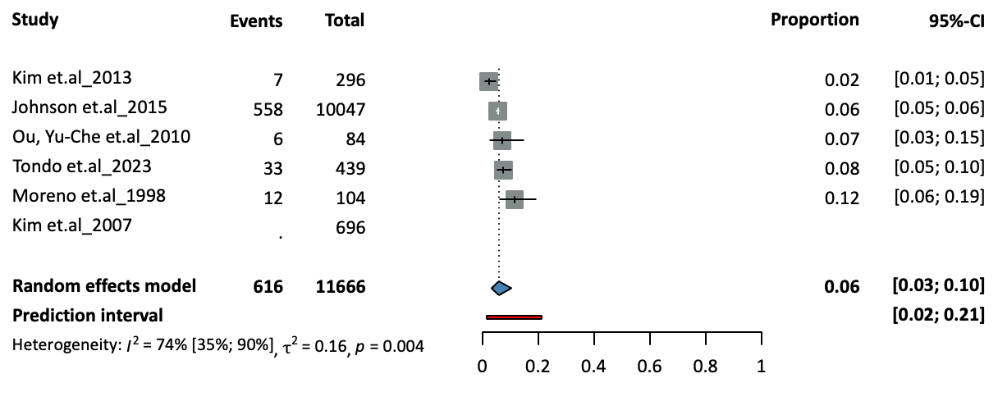


**Figure S4: Fertilization rates in the indirect comparator group**


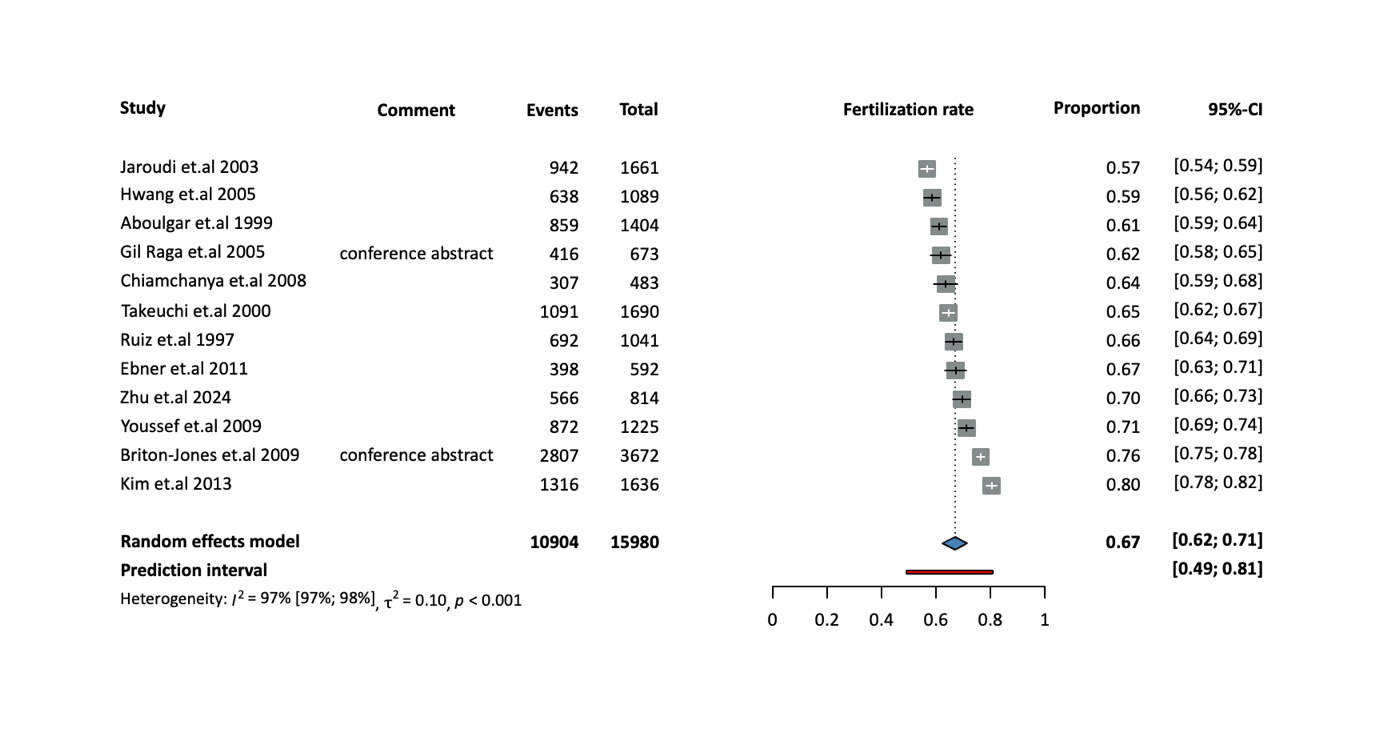


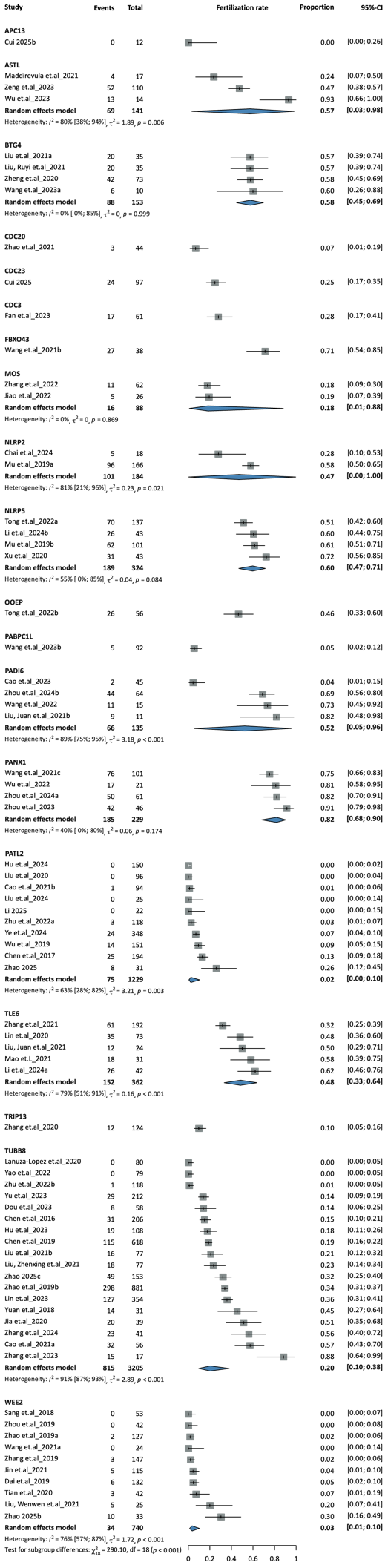


**Figure S5. Forest plot of fertilization rates in patients with different oocyte genetic mutations**

**Figure S6. Funnel plot of TFF rates in patients with WEE2 mutations**

**
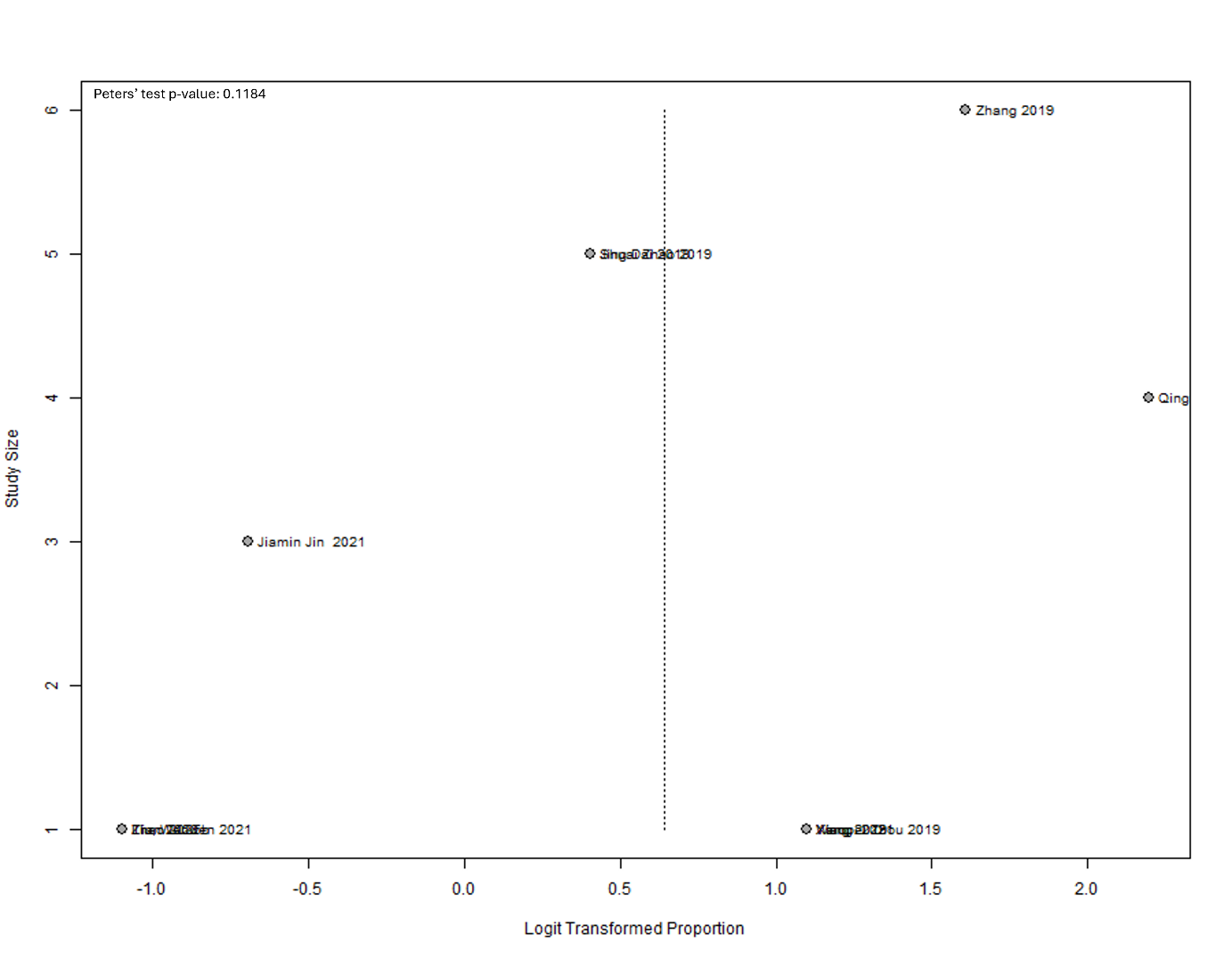
**

**Figure S7. Funnel plot of TFF rates in patients with no genetic testing**

**
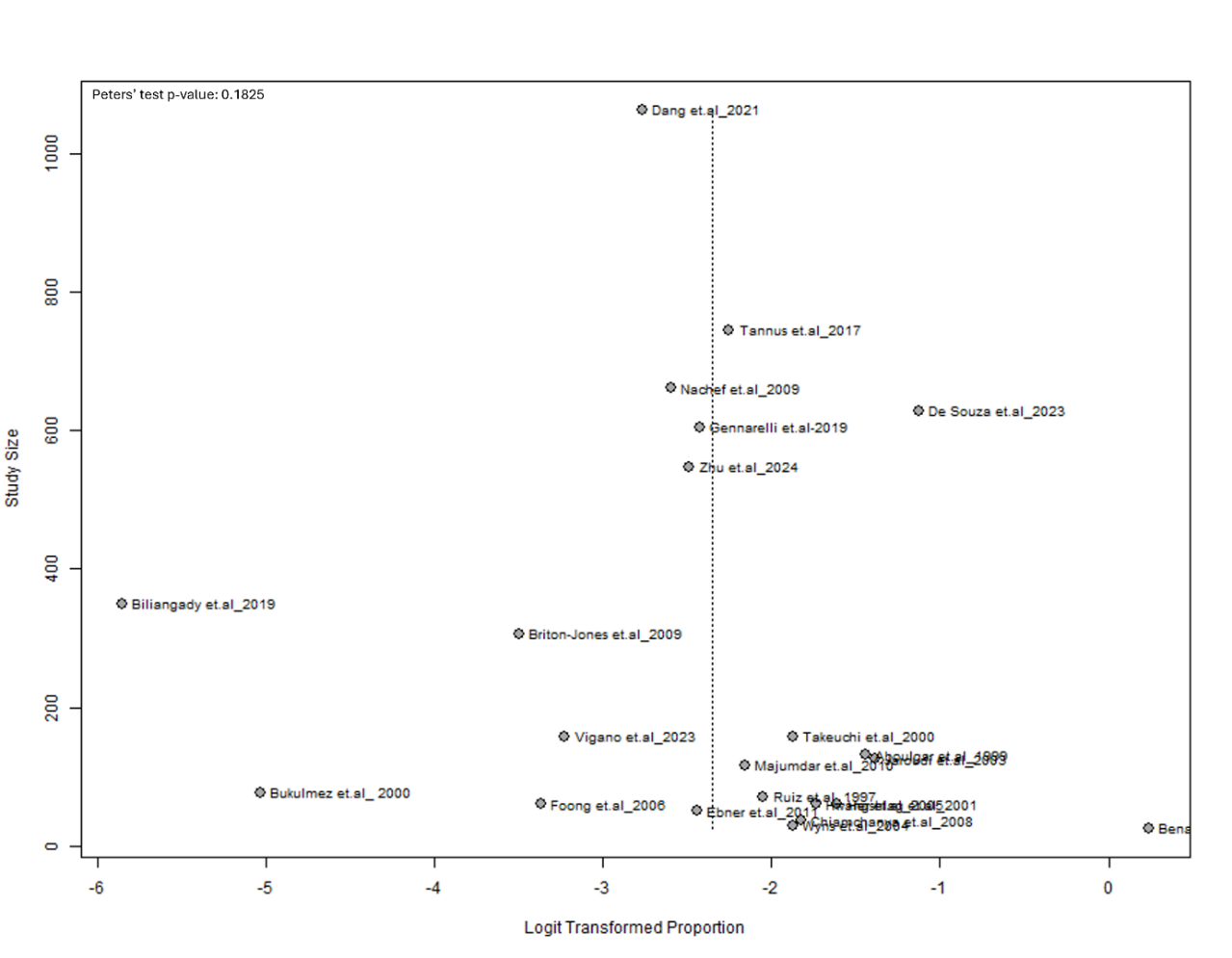
**

**Figure S8. Funnel plot of fertilization rates in patients with no genetic testing**

**
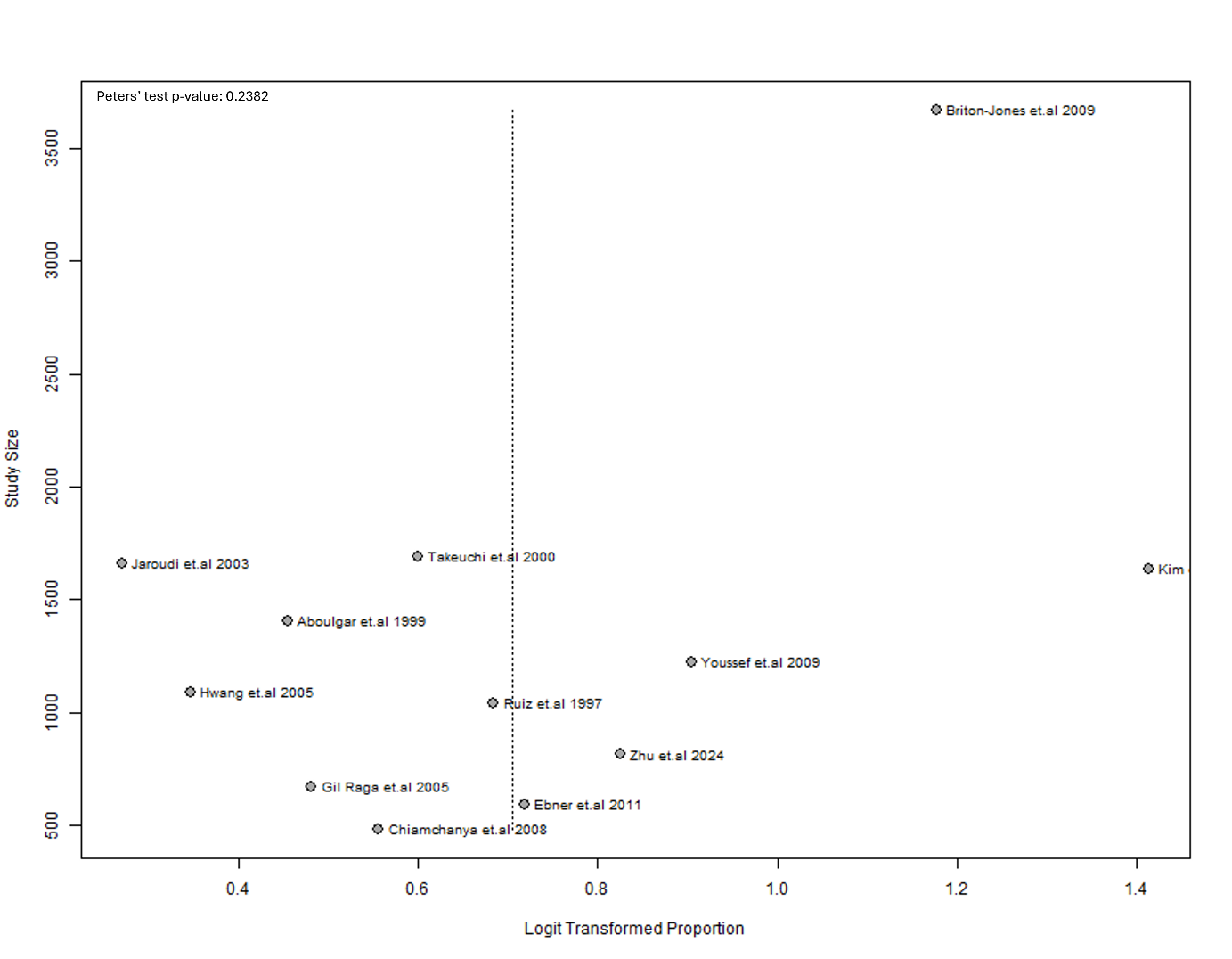
**

**References:**

1. Chen, B., et al., *Novel mutations and structural deletions in TUBB8: expanding mutational and phenotypic spectrum of patients with arrest in oocyte maturation, fertilization or early embryonic development.* Human reproduction (Oxford, England), 2016. **32**(2): p. 457-464.

2. Jin, J., et al., *Novel WEE2 compound heterozygous mutations identified in patients with fertilization failure or poor fertilization.* Journal of Assisted Reproduction and Genetics, 2021. **38**(11): p. 2861-2869.

3. Dai, J., et al., *New biallelic mutations in WEE2: expanding the spectrum of mutations that cause fertilization failure or poor fertilization.* Fertility and Sterility, 2019. **111**(3): p. 510-518.

4. Huang, L., et al., *Novel mutations in PATL2 cause female infertility with oocyte germinal vesicle arrest.* Hum Reprod, 2018. **33**(6): p. 1183-1190.

5. Maddirevula, S., et al., *Female Infertility Caused by Mutations in the Oocyte-Specific Translational Repressor PATL2.* Am J Hum Genet, 2017. **101**(4): p. 603-608.

6. Zhao, S., et al., *Novel WEE2 gene variants identified in patients with fertilization failure and female infertility.* Fertility and Sterility, 2019. **111**(3): p. 519-526.

7. Sang, Q., et al., *Homozygous Mutations in WEE2 Cause Fertilization Failure and Female Infertility.* American Journal of Human Genetics, 2018. **102**(4): p. 649-657.

8. Zhou, X., et al., *Novel compound heterozygous mutations in WEE2 causes female infertility and fertilization failure.* Journal of Assisted Reproduction and Genetics, 2019. **36**(9): p. 1957-1962.

9. Liu, Z., et al., *Novel homozygous mutations in PATL2 lead to female infertility with oocyte maturation arrest.* Journal of assisted reproduction and genetics, 2020. **37**(4): p. 841-847.

10. Chen, B., et al., *Biallelic Mutations in PATL2 Cause Female Infertility Characterized by Oocyte Maturation Arrest.* Am J Hum Genet, 2017. **101**(4): p. 609-615.

11. Lin, T., et al., *Genetic screening and analysis of TUBB8 variants in females seeking ART.* Reproductive BioMedicine Online, 2023. **46**(2): p. 244-254.

12. Zhao, L., et al., *Identification novel mutations in TUBB8 in female infertility and a novel phenotype of large polar body in oocytes with TUBB8 mutations.* Journal of Assisted Reproduction and Genetics, 2020. **37**(8): p. 1837-1847.

13. Hu, T., et al., *Novel variants in TUBB8 gene cause multiple phenotypic abnormalities in human oocytes and early embryos.* Journal of ovarian research, 2023. **16**(1): p. 228-NA.

14. Cao, T., et al., *Two mutations in TUBB8 cause developmental arrest in human oocytes and early embryos.* Reproductive biomedicine online, 2021. **43**(5): p. 891-898.

15. Chen, B., et al., *The comprehensive mutational and phenotypic spectrum of TUBB8 in female infertility.* European Journal of Human Genetics, 2019. **27**(2): p. 300-307.

16. Akbari, M., et al., *A novel variant in TLE6 is associated with embryonic developmental arrest (EDA) in familial female infertility.* Scientific reports, 2022. **12**(1): p. 17664-NA.

17. Cao, Q., et al., *The Recurrent Mutation in PATL2 Inhibits Its Degradation Thus Causing Female Infertility Characterized by Oocyte Maturation Defect Through Regulation of the Mos-MAPK Pathway.* Frontiers in cell and developmental biology, 2021. **9**: p. 628649-628649.

18. Cao, G., et al., *A novel homozygous variant in PADI6 is associate with human cleavage-stage embryonic arrest.* Frontiers in genetics, 2023. **14**: p. 1243230-NA.

19. Chai, M., et al., *A novel homozygous mutation in the NLRP2 gene causes early embryonic arrest.* Journal of assisted reproduction and genetics, 2024. **41**(12): p. 3347-3355.

20. Dong, J., et al., *Novel biallelic mutations in MEI1: expanding the phenotypic spectrum to human embryonic arrest and recurrent implantation failure.* Human reproduction (Oxford, England), 2021. **36**(8): p. 2371-2381.

21. Dou, Q., et al., *Phenotypic variability in two female siblings with oocyte maturation arrest due to a TUBB8 variant.* BMC medical genomics, 2023. **16**(1): p. 271-NA.

22. Fan, H., et al., *Homozygous variants in CDC23 cause female infertility characterized by oocyte maturation defects.* Human genetics, 2023. **142**(11): p. 1621-1631.

23. Hu, H.-Y., et al., *Novel PATL2 variants cause female infertility with oocyte maturation defect.* Journal of assisted reproduction and genetics, 2024. **41**(8): p. 1965-1976.

24. Huang, L., et al., *Novel mutations in NLRP5 and PATL2 cause female infertility characterized by primarily oocyte maturation abnormality and consequent early embryonic arrest.* Journal of assisted reproduction and genetics, 2022. **39**(3): p. 711-718.

25. Jia, Y., et al., *Identification and rescue of a novel TUBB8 mutation that causes the first mitotic division defects and infertility.* Journal of assisted reproduction and genetics, 2020. **37**(11): p. 2713-2722.

26. Jiao, G., et al., *MOS mutation causes female infertility with large polar body oocytes.* Gynecological endocrinology : the official journal of the International Society of Gynecological Endocrinology, 2022. **38**(12): p. 1158-1163.

27. Lanuza-López, M.C., et al., *Oocyte maturation arrest produced by TUBB8 mutations: impact of genetic disorders in infertility treatment.* Gynecological endocrinology : the official journal of the International Society of Gynecological Endocrinology, 2020. **36**(9): p. 829-834.

28. Li, R., et al., *Biallelic Recessive Mutations in <i>TLE6</i> and <i>NLRP5</i> Cause Female Infertility Characterized by Human Early Embryonic Arrest.* Human Mutation, 2024. **2024**(1): p. NA-NA.

29. Lin, J., et al., *Expanding the genetic and phenotypic spectrum of female infertility caused by TLE6 mutations.* Journal of assisted reproduction and genetics, 2020. **37**(2): p. 437-442.

30. Liu, R., et al., *A novel homozygous missense variant in BTG4 causes zygotic cleavage failure and female infertility.* Journal of assisted reproduction and genetics, 2021. **38**(12): p. 1-6.

31. Liu, J., et al., *Two novel mutations in PADI6 and TLE6 genes cause female infertility due to arrest in embryonic development.* Journal of assisted reproduction and genetics, 2021. **38**(6): p. 1551-1559.

32. Liu, Z., et al., *TUBB8 Mutations Cause Female Infertility with Large Polar Body Oocyte and Fertilization Failure.* Reproductive sciences (Thousand Oaks, Calif.), 2021. **28**(10): p. 2942-2950.

33. Liu, W., et al., *A Novel Homozygous Missense Mutation of WEE2 Causes Female Infertility Characterized by Fertilization Failure.* NA, 2021: p. NA-NA.

34. Maddirevula, S., et al., *ASTL is mutated in female infertility.* Human genetics, 2021. **141**(1): p. 1-6.

35. Mao, B., et al., *A novel TLE6 mutation, c.541+1G>A, identified using whole-exome sequencing in a Chinese family with female infertility.* Molecular genetics & genomic medicine, 2021. **9**(8): p. e1743-NA.

36. Mu, J., et al., *Mutations in NLRP2 and NLRP5 cause female infertility characterised by early embryonic arrest.* Journal of medical genetics, 2019. **56**(7): p. 471-480.

37. Okutman, O., et al., *Pathogenic missense variation in PABPC1L/EPAB causes female infertility due to oocyte maturation arrest at the germinal vesicle stage.* Journal of assisted reproduction and genetics, 2024. **41**(2): p. 311-322.

38. Tong, X., et al., *Mutations in OOEP and NLRP5 identified in infertile patients with early embryonic arrest.* Human mutation, 2022. **43**(12): p. 1909-1920.

39. Wang, A., et al., *Clinical exome sequencing identifies novel compound heterozygous mutations of the WEE2 gene in primary infertile women with fertilization failure.* Gynecological Endocrinology, 2021. **37**(12): p. 1096-1101.

40. Wang, W., et al., *FBXO43 variants in patients with female infertility characterized by early embryonic arrest.* Human reproduction (Oxford, England), 2021. **36**(8): p. 2392-2402.

41. Wang, W., et al., *Homozygous variants in PANX1 cause human oocyte death and female infertility.* European journal of human genetics : EJHG, 2021. **29**(9): p. 1396-1404.

42. Wang, X., et al., *A novel homozygous mutation in the PADI6 gene causes early embryo arrest.* Reproductive Health, 2022. **19**(1).

43. Wang, Y., et al., *A novel homozygous C-terminal deletion in BTG4 causes zygotic cleavage failure and female infertility.* Journal of Assisted Reproduction and Genetics, 2023. **40**(1): p. 75-81.

44. Wang, W., et al., *Bi-allelic pathogenic variants in PABPC1L cause oocyte maturation arrest and female infertility.* EMBO molecular medicine, 2023. **15**(6): p. e17177-NA.

45. Weiner, H.S., et al., *Total fertilization failure with in vitro fertilization-intracytoplasmic sperm injection related to WEE2 mutation highlights emerging importance of genetic causes of in vitro fertilization failure.* F S Rep, 2022. **3**(4): p. 355-360.

46. Wu, X.W., et al., *A novel heterozygous variant in PANX1 is associated with oocyte death and female infertility.* Journal of Assisted Reproduction and Genetics, 2022. **39**(8): p. 1901-1908.

47. Wu, H., et al., *Novel biallelic ASTL variants are associated with polyspermy and female infertility: A successful live birth following ICSI treatment.* Gene, 2023. **887**: p. 147745-147745.

48. Xu, Y., et al., *A novel homozygous variant in NLRP5 is associate with human early embryonic arrest in a consanguineous Chinese family.* Clinical genetics, 2020. **98**(1): p. 69-73.

49. Yang, X., et al., *Homozygous missense mutation Arg207Cys in the WEE2 gene causes female infertility and fertilization failure.* Journal of Assisted Reproduction and Genetics, 2019. **36**(5): p. 965-971.

50. Yang, P., et al., *Mutation analysis of tubulin beta 8 class VIII in infertile females with oocyte or embryonic defects.* Clinical Genetics, 2021. **99**(1): p. 208-214.

51. Yao, Z., et al., *Mutation analysis of the TUBB8 gene in primary infertile women with oocyte maturation arrest.* Journal of ovarian research, 2022. **15**(1): p. 38-NA.

52. Ye, Z., et al., *Identification novel mutations and phenotypic spectrum expanding in PATL2 in infertile women with IVF/ICSI failure.* Journal of Assisted Reproduction and Genetics, 2024. **41**(5): p. 1233-1243.

53. Yu, W., et al., *Identification of TUBB8 Variants in 5 Primary Infertile Women with Multiple Phenotypes in Oocytes and Early Embryos.* Reprod Sci, 2023. **30**(4): p. 1376-1382.

54. Zeng, Y., et al., *Bi-allelic variants in ASTL cause abnormal fertilization or oocyte maturation defects.* Human molecular genetics, 2023. **32**(14): p. 2326-2334.

55. Zhang, Z., et al., *Novel mutations in WEE2: Expanding the spectrum of mutations responsible for human fertilization failure.* Clinical Genetics, 2019. **95**(4): p. 520-524.

56. Zhang, Z., et al., *Bi-allelic Missense Pathogenic Variants in TRIP13 Cause Female Infertility Characterized by Oocyte Maturation Arrest.* American journal of human genetics, 2020. **107**(1): p. 15-23.

57. Zhang, M., et al., *Identification of Novel Biallelic TLE6 Variants in Female Infertility With Preimplantation Embryonic Lethality.* Frontiers in Genetics, 2021. **12**.

58. Zhang, Y.-L., et al., *Biallelic variants in MOS cause large polar body in oocyte and human female infertility.* Human reproduction (Oxford, England), 2022. **37**(8): p. 1932-1944.

59. Zhang, J., et al., *A novel compound heterozygous mutation in TUBB8 causing early embryonic developmental arrest.* Journal of assisted reproduction and genetics, 2023. **40**(4): p. 753-763.

60. Zhang, X.-Y., X.-X. Zhang, and L. Wang, *Early embryonic failure caused by a novel mutation in the TUBB8 gene: A case report.* World journal of clinical cases, 2024. **12**(12): p. 2092-2098.

61. Zhao, L., et al., *Identification of Novel Mutations in CDC20: Expanding the Mutational Spectrum for Female Infertility.* Frontiers in Cell and Developmental Biology, 2021. **9**.

62. Zheng, W., et al., *Homozygous Mutations in BTG4 Cause Zygotic Cleavage Failure and Female Infertility.* American journal of human genetics, 2020. **107**(1): p. 24-33.

63. Zhou, J., et al., *A novel heterozygous missense variant of PANX1 causes human oocyte death and female infertility.* Journal of Ovarian Research, 2024. **17**(1).

64. Zhou, J., et al., *Novel variants in PADI6 genes cause female infertility due to early embryo arrest.* Journal of assisted reproduction and genetics, 2024. **41**(12): p. 3327-3336.

65. Zhou, J., et al., *A novel heterozygous variant in PANX1 causes primary infertility due to oocyte death.* Journal of Assisted Reproduction and Genetics, 2023. **40**(1): p. 65-73.

66. Zhu, L., et al., *Oocyte phenotype, genetic diagnosis, and clinical outcome in case of patients with oocyte maturation arrest.* Frontiers in Endocrinology, 2022. **13**.

67. Liu, Z., et al., *Novel splicing mutations in PATL2 and WEE2 cause oocyte degradation and fertilization failure.* Journal of assisted reproduction and genetics, 2024.

68. Sha, Q., et al., *Novel mutations in TUBB8 expand the mutational and phenotypic spectrum of patients with zygotes containing multiple pronuclei.* Gene, 2021. **769**.

69. Tian, Y., et al., *Novel compound heterozygous mutation in WEE2 is associated with fertilization failure: case report of an infertile woman and literature review.* BMC Women's Health, 2020. **20**(1).

70. Wu, L., et al., *Novel mutations in PATL2: expanding the mutational spectrum and corresponding phenotypic variability associated with female infertility.* Journal of Human Genetics, 2019. **64**(5): p. 379-385.

71. Yuan, P., et al., *A novel mutation in the TUBB8 gene is associated with complete cleavage failure in fertilized eggs.* Journal of Assisted Reproduction and Genetics, 2018. **35**(7): p. 1349-1356.

72. Aboulghar, M.A., et al., *Management of long-standing unexplained infertility: A prospective study.* American Journal of Obstetrics and Gynecology, 1999. **181**(2): p. 371-375.

73. Benadiva, C.A., et al., *Intracytoplasmic sperm injection overcomes previous fertilization failure with conventional in vitro fertilization.* Fertility and Sterility, 1999. **72**(6): p. 1041-1044.

74. Biliangady, R., et al., *Are we justified doing routine intracytoplasmic sperm injection in nonmale factor infertility? A retrospective study comparing reproductive outcomes between in vitro fertilization and intracytoplasmic sperm injection in nonmale factor infertility.* Journal of Human Reproductive Sciences, 2019. **12**(3): p. 210-215.

75. Briton-Jones, C.M., et al., *ICSI vs IVF in sibling oocytes on: Fertilization rate, embryo cleavage rate, embryo quality and aneuploidy rates, from patients with primary unexplained infertility and normal semen analysis.* Fertility and Sterility, 2009. **92**(3): p. S36.

76. Bukulmez, O., et al., *Intracytoplasmic sperm injection versus in vitro fertilization for patients with a tubal factor as their sole cause of infertility: a prospective, randomized trial.* Fertil Steril, 2000. **73**(1): p. 38-42.

77. Chiamchanya, C., P. Tor-udom, and N. Gamnarai, *Comparative study of intracytoplasmic sperm injection and in vitro fertilization with high insemination concentration in sibling oocytes in the treatment of unexplained infertility.* Journal of the Medical Association of Thailand, 2008. **91**(8): p. 1155-1160.

78. Dang, V.Q., et al., *Intracytoplasmic sperm injection versus conventional in-vitro fertilisation in couples with infertility in whom the male partner has normal total sperm count and motility: an open-label, randomised controlled trial.* Lancet (london, england), 2021. **397**(10284): p. 1554‐1563.

79. de Souza, L.K., et al., *Total Fertilization Failure: A Single Center Analysis.* Reproductive Sciences, 2024. **31**(3): p. 697-703.

80. Ebner, T., et al., *Assisting in vitro fertilization by manipulating cumulus-oocyte-complexes either mechanically or enzymatically does not prevent IVF failure.* Journal of the Turkish German Gynecology Association, 2011. **12**(3): p. 135-139.

81. Foong, S.C., et al., *A prospective randomized trial of conventional in vitro fertilization versus intracytoplasmic sperm injection in unexplained infertility.* Journal of Assisted Reproduction and Genetics, 2006. **23**(3): p. 137-140.

82. Gennarelli, G., et al., *ICSI Versus Conventional IVF in Women Aged 40 Years or More and Unexplained Infertility: A Retrospective Evaluation of 685 Cycles with Propensity Score Model.* J Clin Med, 2019. **8**(10).

83. Gil Raga, F., et al., *Analysis of the results of cycles of IVF-ICSI in no pregnant couples alter four intrauterine inseminations.* Revista Iberoamericana de Fertilidad y Reproduccion Humana, 2005. **22**(2): p. 113-120.

84. Hershlag, A., et al., *In vitro fertilization-intracytoplasmic sperm injection split: An insemination method to prevent fertilization failure.* Fertility and Sterility, 2002. **77**(2): p. 229-232.

85. Hwang, J.L., et al., *IVF versus ICSI in sibling oocytes from patients with polycystic ovarian syndrome: a randomized controlled trial.* Hum Reprod, 2005. **20**(5): p. 1261-5.

86. Jaroudi, K., et al., *Intracytoplasmic sperm injection and conventional in vitro fertilization are complementary techniques in management of unexplained infertility.* Journal of Assisted Reproduction and Genetics, 2003. **20**(9): p. 377-381.

87. Johnson, L., M.D. Sammel, and A. Dokras, *Intracytoplasmic sperm injection reduces total failed fertilization rate but does not improve pregnancy or live birth rates in unexplained infertility: Analysis of over 20,000 cycles from the SART database.* Fertility and Sterility, 2015. **104**(3): p. e66.

88. Kim, J.Y., et al., *Can intracytoplasmic sperm injection prevent total fertilization failure and enhance embryo quality in patients with non-male factor infertility?* European Journal of Obstetrics and Gynecology and Reproductive Biology, 2014. **178**: p. 188-191.

89. Kim, H.H., et al., *Use and outcomes of intracytoplasmic sperm injection for non-male factor infertility.* Fertility and Sterility, 2007. **88**(3): p. 622-628.

90. Majumdar, G., R. Gaur, and A. Majumdar, *Post swim-up sperm count as a predictor for fertilization-failure in patients undergoing IVF with normozoospermia.* Journal fur Reproduktionsmedizin und Endokrinologie, 2010. **7**(4): p. 330.

91. Moreno, C., et al., *Intracytoplasmic sperm injection as a routine indication in low responder patients.* Human Reproduction, 1998. **13**(8): p. 2126-2129.

92. Nachef, S., et al., *Efficacy of rescue ICSI after conventional IVF failure.* Jornal Brasileiro de Reproducao Assistida, 2009. **13**(3): p. 18-20.

93. Ou, J.P., et al., *Analysis of the clinical outcomes of rescue ICSI after total fertilization failure in 115 conventional IVF cycles.* Human Reproduction, 2010. **25**: p. i131.

94. Ruiz, A., et al., *The role of in vitro fertilization and intracytoplasmic sperm injection in couples with unexplained infertility after failed intrauterine insemination.* Fertility and Sterility, 1997. **68**(1): p. 171-173.

95. Takeuchi, S., et al., *In vitro fertilization and intracytoplasmic sperm injection for couples with unexplained infertility after failed direct intraperitoneal insemination.* Journal of Assisted Reproduction and Genetics, 2000. **17**(9): p. 515-520.

96. Tannus, S., et al., *The role of intracytoplasmic sperm injection in non-male factor infertility in advanced maternal age.* Human Reproduction, 2017. **32**(1): p. 119-124.

97. Tondo, F., et al., *Total Fertilization Failure after conventional IVF: it is not the end of the story.* Human Reproduction, 2023. **38**: p. i307.

98. Viganò, P., et al., *Conventional IVF performs similarly in women with and without endometriosis.* Journal of Assisted Reproduction and Genetics, 2023. **40**(3): p. 599-607.

99. Wyns, C., et al., *IVF and ICSI outcome in couples with unexplained infertility: A randomized study of 60 cases.* Jornal Brasileiro de Reproducao Assistida, 2004. **8**(5): p. 16-24.

100. Youssef, H.M., et al., *Fertilization and live birth rates followeing conventional IVF versus ICSI in non male factor: A prospective randomized study using sibling oocytes.* Fertility and Sterility, 2009. **92**(3): p. S225.

101. Li, Z., et al., *Fertilization of IVF/ICSI using sibling oocytes from couples with subfertile male or unexplained infertility.* J Huazhong Univ Sci Technolog Med Sci, 2004. **24**(4): p. 365-8, 384.

102. Zhu, S., et al., *Intracytoplasmic sperm injection compared with in vitro fertilisation in patients with non-male factor infertility with low oocyte retrieval: a single-centre, retrospective cohort study.* BMJ Open, 2024. **14**(11): p. e080688.

103. Cui, Y., et al., *Infertile females with biallelic mutations in APC/C genes are characterized by oocyte or early embryo defects.* J Assist Reprod Genet, 2025. **42**(5): p. 1587-1599.

104. Li, H., et al., *Identification of a PATL2 missense variant (c.877G>T) disrupting canonical splicing and contributing to female infertility.* Front Genet, 2025. **16**: p. 1611138.

105. Zhao, H., et al., *Identification of novel variants and expansion of the phenotypic spectrum in PATL2, WEE2, and TUBB8 associated with human early embryonic arrest.* J Assist Reprod Genet, 2025. **42**(6): p. 1961-1973.
